# Reordering Frustration in Enantiomer-Enriched Solid Solutions of Ionic Plastic Crystals for Proton-Conducting Materials Design

**DOI:** 10.1021/acs.chemmater.6c00948

**Published:** 2026-06-27

**Authors:** Andrea Vitale, Wenjing Chen, Antunes Staffolani, Massimo Marcaccio, Elisabetta Venuti, Tommaso Salzillo, Simone D’Agostino

**Affiliations:** † Department of Chemistry “Giacomo Ciamician”, 9296 The University of Bologna, Via P. Gobetti 85, 40129 Bologna, Italy; ‡ Department of Industrial Chemistry “Toso Montanari”, The University of Bologna, Via P. Gobetti 85, 40129 Bologna, Italy; § ENERCube, Centro Ricerche Energia, Ambiente e Mare, Centro Interdipartimentale per la Ricerca Industriale Fonti Rinnovabili, Ambiente, Mare ed Energia (CIRI-FRAME)—Alma Mater Studiorum University of Bologna, Viale Ciro Menotti, 48, 48122 Marina di Ravenna, Italy

## Abstract

A series of triflate (TFO^–^) salts with various globular cations, namely, the achiral 3-quinuclidonium [QHco]^+^, the racemic (3)-hydroxyquinuclidinium S/R-[QH]^+^, and the enantiopure (3)-hydroxyquinuclidinium R-[QH]^+^, have been synthesized and characterized via a combination of X-ray diffraction and microcalorimetric techniques. Despite the similarity among their components, the three salts displayed different behaviors. At room temperature, the achiral [QHco]­TFO crystallized in an ordered phase, and no transition to the plastic phase was detected. In contrast, the racemic and enantiopure salts, namely, S/R-[QH]­TFO and R-[QH]­TFO at room temperature crystallized as disordered phases; only the latter underwent a transition from a plastic phase to an ordered one upon cooling, whereas the former remained permanently disordered, even at the lowest accessible temperature. The formation of solid solutions was investigated for the pair S/R-[QH]­TFO/R-[QH]­TFO, S/R-[QH]­TFO/[QHco]­TFO and R-[QH]­TFO/[QHco]­TFO and found to be successful only in the first case, whereas it led to physical mixtures in the other two. Notably, the S/R-[QH]­TFO/R-[QH]­TFO solid solution retained its disordered phase over a wide temperature range, a behavior attributed to a phenomenon we describe as “reordering frustration”. Raman spectroscopy complemented and confirmed the structural analysis, and electrochemical impedance spectroscopy was successfully applied to study proton conductivity associated with temperature variations in the pure salts and S/R-[QH]­TFO/R-[QH]­TFO solid-solutions under inert and dry conditions.

## Graphical abstract

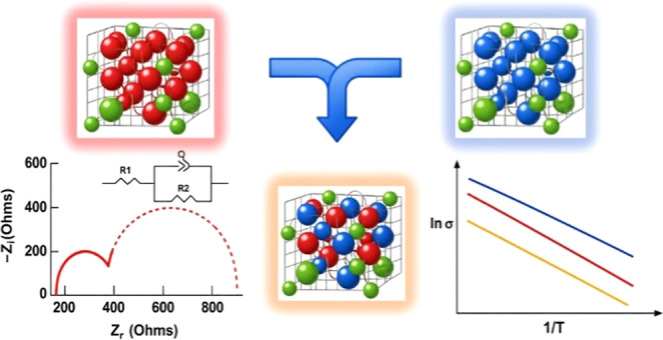

## Introduction

Proton-conducting materials play a crucial role in advanced technologies such as fuel cells, sensors, batteries, and electrochemical devices for energy production and storage.^1–4^

Recent studies have identified plastic crystals (PCs) and ionic plastic crystals (IPCs) as promising candidates for the development of advanced solid-electrolytes^5–8^ or as dopants for Nafion-based membranes.^8–13^

As electrolyte materials, IPCs offer several key advantages, including nonflammability, low vapor pressure, plasticity, and excellent thermal stability. Importantly, they display intrinsic ionic conductivity, enhanced by their dynamic nature.^8,14^ Beyond their applications in electrochemical systems, their peculiar features make them potential candidates for diverse applications such as barocalorics,^15–17^ ferroelectrics,^18–20^ and solid–solid phase change materials (SS-PCM) for thermal energy storage applications.^21–23^

The unique traits of PCs and IPCs stem from their structure. These molecular compounds feature a mesophase, specifically, a plastic crystal phase, between solid and liquid states.^20,24^ In this phase, also known as rotary phase, molecules retain a crystalline lattice with long-range order but undergo rapid, isotropic rotational motion, causing orientational disorder like in liquids. Moreover, they exhibit several unique features that are different from regular crystals, such as malleability (plastic deformation without a fracture upon application of uniaxial pressure, as in waxes), self-diffusion, and large entropy changes in solid–solid phase transitions in response to suitable stimuli like temperature and pressure.^25–27^ These transitions can lead to ordered or glassy crystals,^24^ as depicted in Scheme 1.

**Scheme 1:**
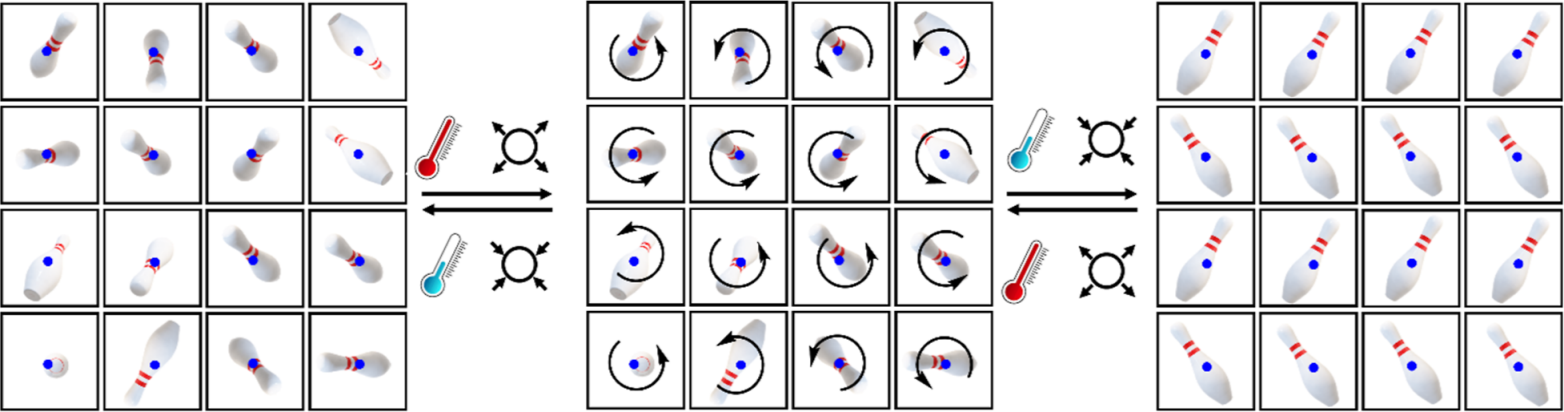
Representation of the Possible Solid–Solid Transitions in Response to Temperature and Pressure: A Statically Disordered Glassy Crystal (Left), a Dynamically Disordered Plastic Crystal (Middle), and an Ordered Crystal (Right)^a^

a Chemical species and center of mass represented by skittles and blue spheres, respectively, arrows indicate re-orientational motion around center of mass.

Usually, PCs and IPCs consist of molecules and ions with nearly spherical or disc like shapes,^8,24,28^ that can easily rotate in crystals. However, predicting effective ionic combinations and transition temperatures remains challenging, making the exploration of molecular and structural factors crucial for advancing design strategies in this field.

Previous studies have demonstrated plastic phase behavior in molecular salts containing the enantiopure cation (3)-hydroxyquinuclidinium with various anions,^7,29^ and by leveraging crystal engineering principles,^30,31^ we have also explored their solid-solutions^32,33^ with different anions to fine-tune the transition temperature.^32,34^

In this study, we present our findings on a series of triflate salts synthesized with different globular cations, including achiral 3-quinuclidonium [QHco]^+^, racemic (3)-hydroxyquinuclidinium S/R-[QH]^+^, and enantiopure (3)-hydroxyquinuclidinium R-[QH]^+^ (Chart 1). Additionally, the triflate anion (TFO) was chosen to mimic the sulfonic acid moiety of Nafion.

**Chart 1:**
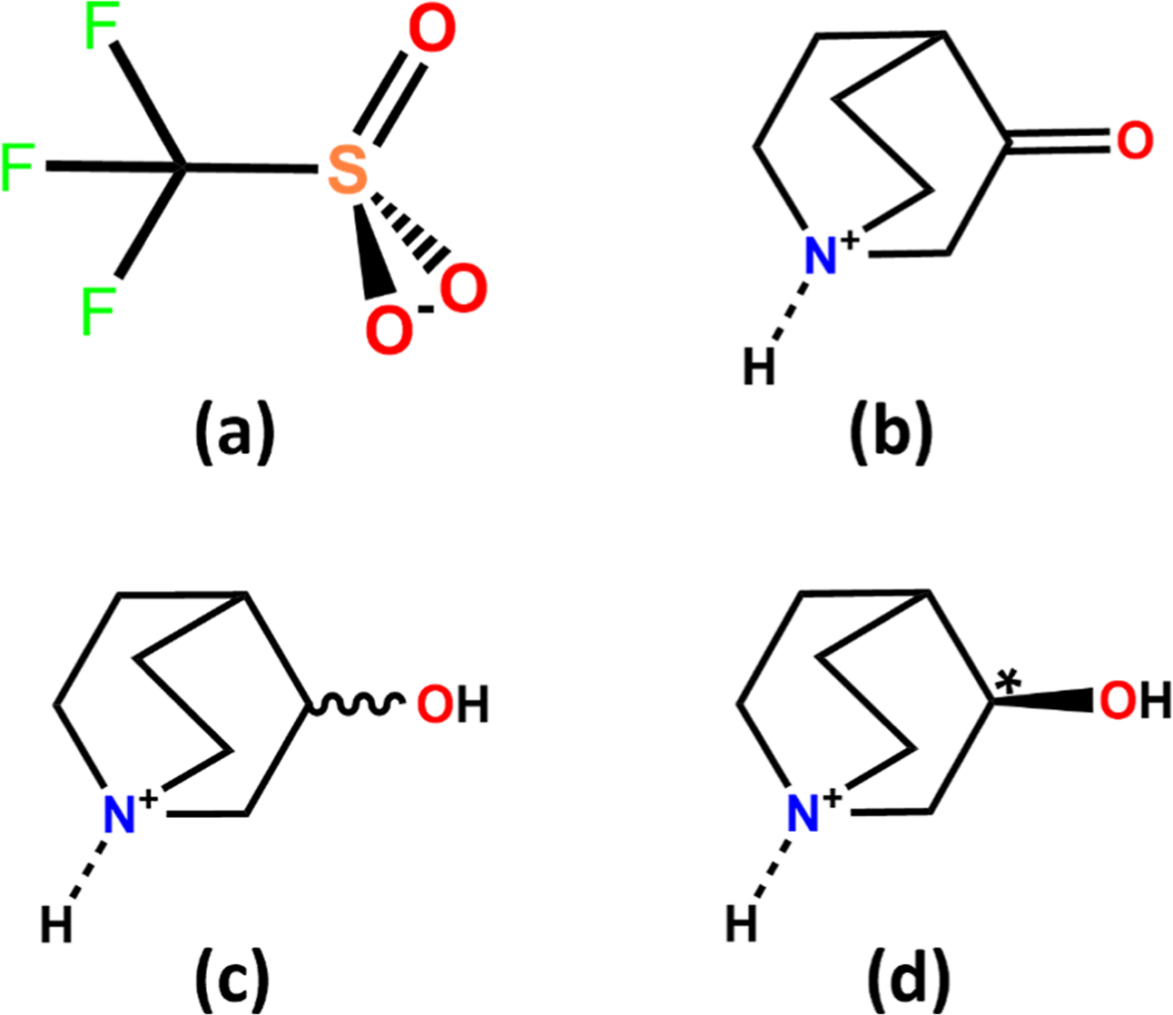
Molecular Structures of the Ionic Building Blocks Chosen as Components for the Preparation of IPCs: (a) The Triflate Anion, (b) the Achiral 3-Quinuclidonium [QHco]^+^, (c) The Racemic Hydroxyquinuclidinium S/R-[QH]^+^, and (d) The Enantiopure R-Hydroxyquinuclidinium R-[QH]^+^ Cations; *Indicates the Chiral Carbon Atom

The crystalline salts were structurally characterized using X-ray diffraction (XRD) techniques, and their plastic behavior was examined through microcalorimetric analyses. Notably, both the enantiopure R-[QH]­TFO and the racemic S/R-[QH]­TFO salts exhibited a plastic phase at room temperature. However, while the enantiopure form undergoes a phase transition to a semiordered phase upon cooling, the racemic counterpart remains in a permanently disordered state. Due to their isomorphism in the plastic phase at RT, as they share the same unit cell parameters, despite one being racemic and the other enantiopure, the potential for solid-solution formation was also explored using a combination of Raman spectroscopy and electrochemical impedance spectroscopy (EIS) to evaluate how composition affects phase transition behavior and conduction properties.

## Experimental Section

### Synthesis

All reagents and reactants were purchased from Sigma-Aldrich and used without any further purification. Bidistilled water and reagent grade solvents were used. The salts [QHco]­TFO, S/R-[QH]­TFO, and R-[QH]­TFO were obtained by the anion exchange reaction between the halide abstraction reagent, silver triflate (AgSO_3_CF_3_), and the corresponding hydrochloride salts of the quinuclidinium derivative. In a typical reaction, 100 mg (0.5 mmol) of AgSO_3_CF_3_ was dissolved in a beaker with 3 mL of water and left under stirring at RT. Next, the stoichiometric amount of hydrochloride salts of the quinuclidinium derivative was dissolved in another beaker with ca. 2 mL of water. The resulting solution was added dropwise to the solution of AgSO_3_CF_3_ and left under stirring for ca. 2 h. Then, the precipitated AgCl was filtered off, and the resulting solution was left to slowly evaporate to afford crystalline materials suitable for single-crystal analysis.

### Atomic Force Microscopy (AFM)

AFM imaging and analysis were performed in air with a Digital NanoScope 3D Multimode microscope (Veeco, USA) using phosphorus n-doped Silicon probes (spring constant, 10–40 N/m; resonance frequency 290–330 kHz; nominal tip radius <10 nm) and operating either in tapping or contact mode. Samples for the AFM experiments were prepared by immobilizing the crystals on quartz glass slide by a thin film of fast methacrylate glue and performing the experiments few minutes after the crystals were fixed onto the substrate. The images were obtained by optimizing the set point tapping amplitude with respect to the free amplitude of the probe, near the resonance frequency of the probe itself, as reported elsewhere.^35^ The force curves were obtained by moving vertically the tip, through approaching and retracting the tip itself on the crystal surface, and starting the movement of the probe from a height of, at least, 500 nm away from the surface and recording the deflection. The spring force constant was calibrated both by thermal noise method and the Sader method^36,37^ providing a value of 21 N/m for the AFM tip used. All the data collected comprised several indentation measurements (at least 10) performed in different points with a distance between each other of at least 500 nm. Concerning the analysis of the force curves, all the data have been elaborated following the Hertz model^38,39^ and the procedure by Olivier and Pharr.^40^ Prior to investigating the force curves of the single crystal samples, the behavior of the supporting glass surface was examined and the corresponding force curve diagram is reported in the Supporting Information. As expected, it shows a typical behavior of a solid with a nondeformable surface, with a very steep increase of the force upon decreasing the tip–surface distance in the contact region. The retraction curve is characterized by clear evidence of adhesion forces. Moreover, in the same diagram the interpolation line (black dashed line) of the retraction curve is also reported as a measure of the load/displacement slope, that is related to stiffness modulus.

### X-ray Diffraction (XRD)

Single-crystal data for all compounds were collected on an Oxford X’Calibur S CCD diffractometer equipped with a graphite monochromator (Mo Kα radiation) and with a cryostat Oxford CryoStream800. Structures were solved with SHELXT by intrinsic phasing^41^ and refined on *F* ^2^ with SHELXL^42^ implemented in the Olex2 software^43^ by full-matrix least-squares refinement. All non-hydrogen atoms were refined anisotropically and the rigid-body RIGU restraints was applied.^44^ H_OH_ and H_NH_ atoms were directly located, when possible, while H_CH_ atoms were added in calculated positions and refined riding on their respective carbon atoms. Data collection and refinement details are listed in Tables SI1a,b. The Mercury^45^ program was used for molecular graphics and calculation of intermolecular interactions. Crystal data can be obtained free of charge via http://www.ccdc.cam.ac.uk/conts/retrieving.html (or from the Cambridge Crystallographic Data Centre, 12 Union Road, Cambridge CB21EZ, UK; fax: (+44)­1223-336-033; or e-mail: deposit@ccdc.cam.ac.uk); CCDC numbers 2541507–2541515.

For ϕ-scan experiments, a fresh single crystal specimen of R-[QH]­TFO was selected and mounted on the diffractometer. Goniometer angles (θ, *k*, ω, ϕ) were set at 0° and detector distance at 43 mm, and then ϕ was moved by 1° during the exposure time (20 s). Unit cell determinations at RT and 100 K were performed and corresponded to those of the salt R-[QH]­TFO at RT and 250 K, respectively.

For phase identification and variable-temperature X-ray powder diffraction purposes, measurements were performed on a PANalytical X’Pert Pro automated diffractometer equipped with an X’Celerator detector in Bragg–Brentano geometry, using Cu Kα radiation without a monochromator in the 2θ range 5–40° (continuous scan mode, step size 0.0167°, counting time 19.685 s, Soller slit 0.04 rad, antiscatter slit 1/2, divergence slit 1/4, 40 mA × 40 kV) and with an Anton-Paar TTK 450+ LNC. The program Mercury^45^ was used for the calculation of powder XRD patterns based on single-crystal data collected in this work. Chemical and structural identities between bulk materials and single crystals were always verified by comparing experimental and calculated powder diffraction patterns. For Pawley refinement and structural solution from powder data purposes, diffractograms in the 2θ range 3–70° (step size, 0.026°; time/step, 200 s; 0.02 rad soller; *V* × *A* 40 × 40) were collected on a Panalytical X’Pert PRO automated diffractometer equipped with a PIXcel detector in transmission geometry (capillary spinner), using Cu Kα radiation (λ = 1.5418 Å) without monochromator in the 2θ range 3–70° (continuous scan mode, step size 0.0260°, counting time 889.70 s, Soller slit 0.02, antiscatter slit 1/4, divergence slit 1/4, 40 mA*40 kV). Six patterns were recorded and summed to enhance the signal-to-noise ratio. For Pawley refinements, diffractograms were analyzed with the software TOPAS4.^46^ A shifted Chebyshev function with 8 parameters was used to fit the background; see Supporting Information for difference pattern plots and the associated Figures of Merit.

### Thermal Analyses

Thermogravimetric analyses (TGA) were performed with a PerkinElmer TGA-7. The samples were contained in a platinum crucible and heated under nitrogen flow (20 cm^3^ min^–1^) at a rate of 5 °C min^–1^ up to decomposition. Sample weights were in the range 5–10 mg. Differential scanning calorimetry (DSC) measurements were performed with a PerkinElmer DSC-7 equipped with a PII intracooler. Temperature and enthalpy calibrations were performed using high-purity standards (*n*-decane, benzene, and indium). Heating of the aluminum open pan containing the samples (3–5 mg) were carried out at 5 °C min^–1^ under a *N*_2_(g) atmosphere. Entropy changes were estimated by dividing the enthalpy changes by the transition temperature. Hot-stage experiment was carried out using a Linkam TMS94 device connected to a Linkam LTS350 platinum plate and equipped with polarizing filters. Images were collected with a NIKON DS FI3 camera from an Olympus BX41 stereomicroscope.

### Thermal Expansion Coefficients Determination

The linear thermal expansion coefficients of [QHco]­TFO and R-[QH]­TFO were calculated using the PASCal (Principal Axis Strain Calculator) program.^47^ For this purpose, crystal structures of both compounds were collected at increasing temperatures, and the refined unit cell parameters (see Tables SI1a,b) were used as input. Results are presented in Table 1.

**Table 1:** Coefficients of Linear Thermal Expansion α for the *a*, *b*, and *c* Crystallographic Axes and Unit Cell Volume of [QHco]­TFO and R-[QH]­TFO

	α_a_ (M·K^–1^)	α_b_ (M·K^–1^)	α_c_ (M·K^–1^)	*V* (M·K^–1^)
[QHco]TFO	–27(2)	74(7)	160(3)	211(8)
R-[QH]TFO	25(3)	53(2)	114(9)	195(14)

### Raman Spectroscopy

Raman measurements were performed with a custom-built system based on a 640 mm focal length HORIBA T64000 triple grating spectrometer. The ultralow-frequency (ULF) range, extending into the THz region (<150 cm^–1^), was accessed via the double subtractive + single additive configuration. Spectra were recorded with the excitation wavelength of the 514.5 nm line of an Ar^+^ gas laser, focused on the sample by an optical confocal microscope Olympus BX40 equipped with a 20× objective, providing a nominal spatial resolution of approximately 5 μm. The combination of the 514 nm excitation line, the use of a plane aberration corrected holographic grating with 1800 gr mm^–1^, coupled with a 1024 × 256 pixels CCD detector enabled a maximum spectral resolution of 1.5 cm^–1^. Variable temperature Raman spectra were collected using a MHCS600-P vacuum hot and cold stage coupled with MTDC600 temperature control, allowing measurements between 150 °C and −190 °C in atmosphere conditions, with cooling achieved with liquid nitrogen.

### Electrochemical Impedance Spectroscopy

The ionic conductivity was measured by electrochemical impedance spectroscopy (EIS) in the temperature range 30–140 °C with (10 °C steps). A VSP-3e (Bio-Logic SAS, Seyssinet-Pariset, France) potentiostat/galvanostat/frequency analyzer was used for the acquisition of the AC-spectra. The measurements were performed in a two-electrodes configuration with stainless steel 316L blocking electrodes. The electrolyte powders were dried at 100 °C under vacuum for 1 h (Büchi B-585, Cornaredo, Italy) before transferring them inside a dry room with dew point below −40 °C (Il Disgelo srl, Torino, Italy). The electrolyte pellets were prepared with *Ø* = 13 mm by a hydraulic press; each one was prepared by using ≈100 mg of electrolyte material at the pressure of 1000 psi (≈70 kg cm^–2^). To ensure proper electronic contact, each side of the pellets were coated with an electronically conductive Ag-paste (Elettro’340 Argento conductive paint). 2032-coin cells were used for this study with two 1 mm spacers and crimped with a pressure of 500 psi. The cell assembly procedure was performed inside an Ar-filled glovebox with H_2_O and O_2_ levels <0.1 ppm (MBraun LabMaster Pro ECO, Garching, Germany). The AC-spectra were acquired by applying a potential perturbation Δ*E* = 50 mV at the open circuit potential, in the frequency range of 1 MHz < ν < 1 Hz and with 10 points per decade. EIS measurements were performed at different temperatures, starting from 30 °C up to 140 °C with 10 °C intervals by using a programmable oven (Pol-Eko, Wodzisław Śląski, Poland). The cells were allowed to reach a thermal equilibration 30 min before the AC-impedance acquisition.

The EIS spectra were fit by using an equivalent circuit model (ECM) consisting of a parallel made of a resistor and a constant phase element, connected in series with a constant phase element i.e., (RQ)­Q in Boukamp’s notation.^48^ The fit procedure was done by using the Python software DearEis^49^ and optimized to reach a χ^2^ < 10^–4^.

## Results and Discussion

The crystalline molecular salts of this study, featuring the globular cations 3-quinuclidonium, [QHco]^+^, racemic hydroxyquinuclidinium, S/R-[QH]^+^, and enantiopure R-hydroxyquinuclidinium, R-[QH]^+^, with triflate (TFO^–^) as the counterion, were synthesized by reacting commercially available hydrochloride salts with silver­(I) triflate, which served as a halide extraction reagent and left to slowly evaporate at RT (see the Experimental Section for details).

Through visual inspection, the three crystallized salts displayed distinct appearances as well as different mechanical properties upon touch. The achiral compound [QHco]­TFO appeared as a typical brittle crystalline solid, crumbling easily when pressed with a needle. In contrast, both the enantiopure and racemic salts, R-[QH]­TFO and S/R-[QH]­TFO, yielded unusual crystalline samples that conformed to the vessel’s shape and exhibited a glassy appearance with a caramel-like texture. Unlike the achiral crystals, these did not crumble under pressure from a needle; instead, they deformed while retaining their integrity. Figure 1 compares the contrasting mechanical behavior of the achiral and enantiopure single crystals, while AFM was used to quantify their mechanical properties.

**Figure 1:**
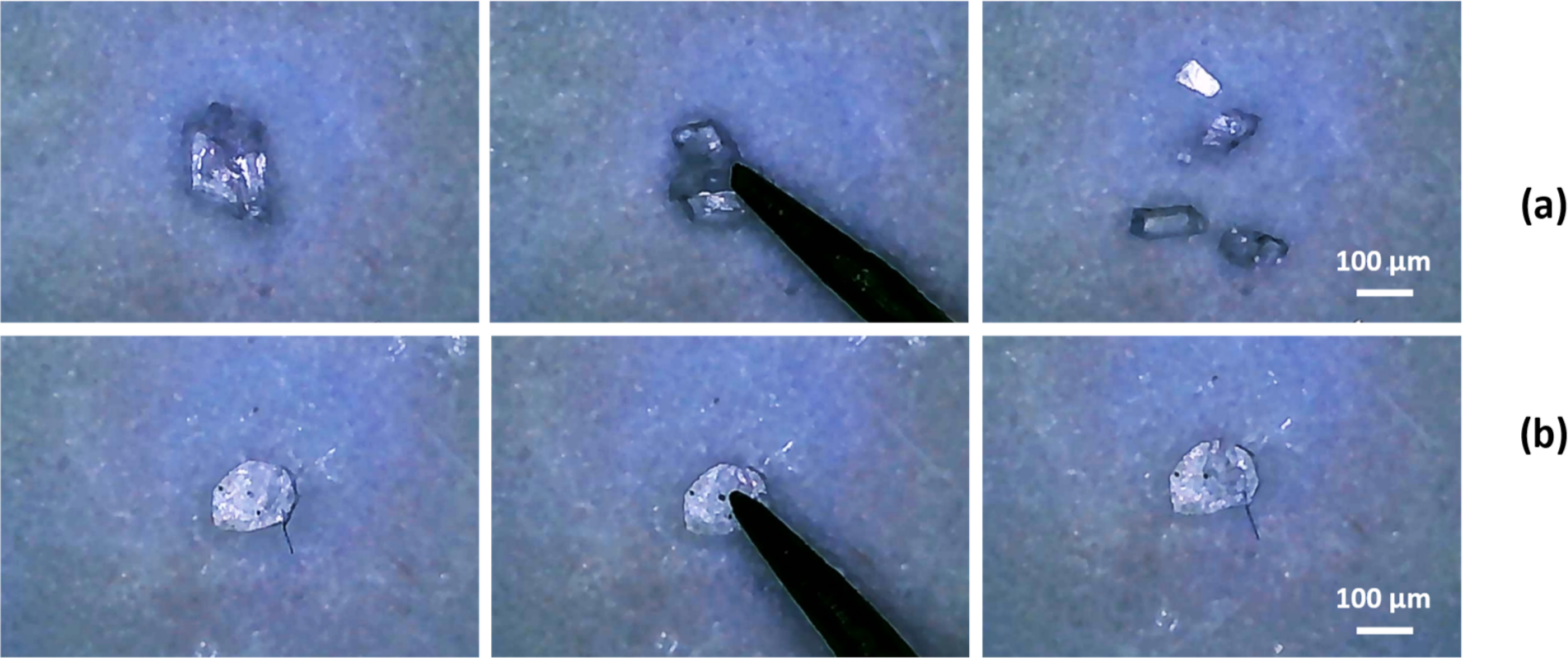
Comparison of the mechanical behavior of: (a) [QHco]­TFO and (b) R-[QH]­TFO. The achiral salt forms brittle crystals that crumble under pressure, while the enantiopure one forms a softer crystal that deforms rather than breaks.

In the case of the achiral crystal [QHco]­TFO, that is a brittle material, the corresponding force curves are similar to that of the glass substrate, on which it is immobilized, although the [QHco]­TFO shows a lower slope of the interpolated loading and unloading region (Figure 2a). This indicates that the stiffness of the material is lower than the quartz glass (see Figure SI1). Expectedly, the adhesion phenomenon is observed as evidenced by the presence of the characteristic jump-off-contact. Within the range of loading force applied it is possible to observe that this crystal behaves as an incompressible material. On the other hand, investigation of the enantiopure crystal R-[QH]­TFO under the same experimental conditions and following the same sample preparation procedure shows a rather different behavior, as expected for a plastic material. Concerning the approaching curve (blue trace in Figure 2b) the contact point is not easily identifiable as in the case of [QHco]­TFO, since the material is deformable under the pressure applied by the tip and thus its surface is modified accordingly. Thus, the retraction curve, i.e., the unloading process during the tip withdraw, can be used to calculate the value of the elastic modulus for such a material, through the Hertz model,^38,39^ with the procedure followed by Oliver and Pharr^40^ for which the slope of the force curve is directly proportional to the effective Young’s modulus that can be obtained. Thus, the effective Young’s modulus for [QHco]­TFO is estimated to be 0.4 MPa while for the R-[QH]­TFO it is about one-order of magnitude lower with a value of 0.07 MPa, thus confirming and supporting the observations gathered by visual inspection about the rather different mechanical characteristics of the two crystals.

**Figure 2:**
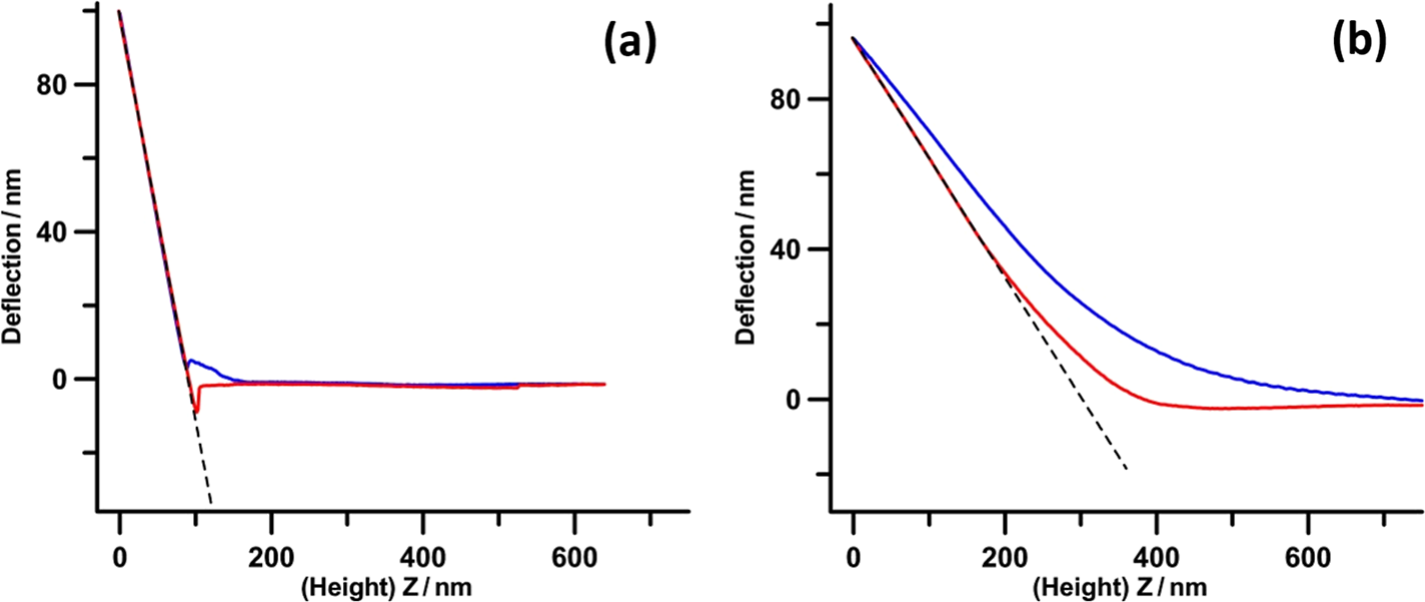
Force–distance (deflection vs tip travel, *z*-scan) curves recorded on single-crystal samples of (a) [QHco]­TFO and (b) R-[QH]­TFO, immobilized on a quartz glass slide. The approach is shown in blue and the retraction in red; the black dashed line represents the linear interpolation of the initial segment of the retraction curve with equation Defl = −1.761·*Z* + 98.10 for (a) [QHco]­TFO and Defl = −0.3175·*Z* + 95.88 for (b) R-[QH]­TFO.

Structural analysis performed at RT on [QHco]­TFO shows that the compound crystallizes in the monoclinic system and with the *P*2_1_/*n* space group (see Table SI1a for details). As reported in Figure 3, the salt forms discrete anion/cation pairs interacting through a bifurcated charge-assisted hydrogen bond [N–H···O = 3.019(5), 3.192(6) Å], while the carbonyl group is engaged only in weak bifurcated interactions with neighboring cations [C–H···O = 3.264(5), 3.459(5) Å].

**Figure 3:**
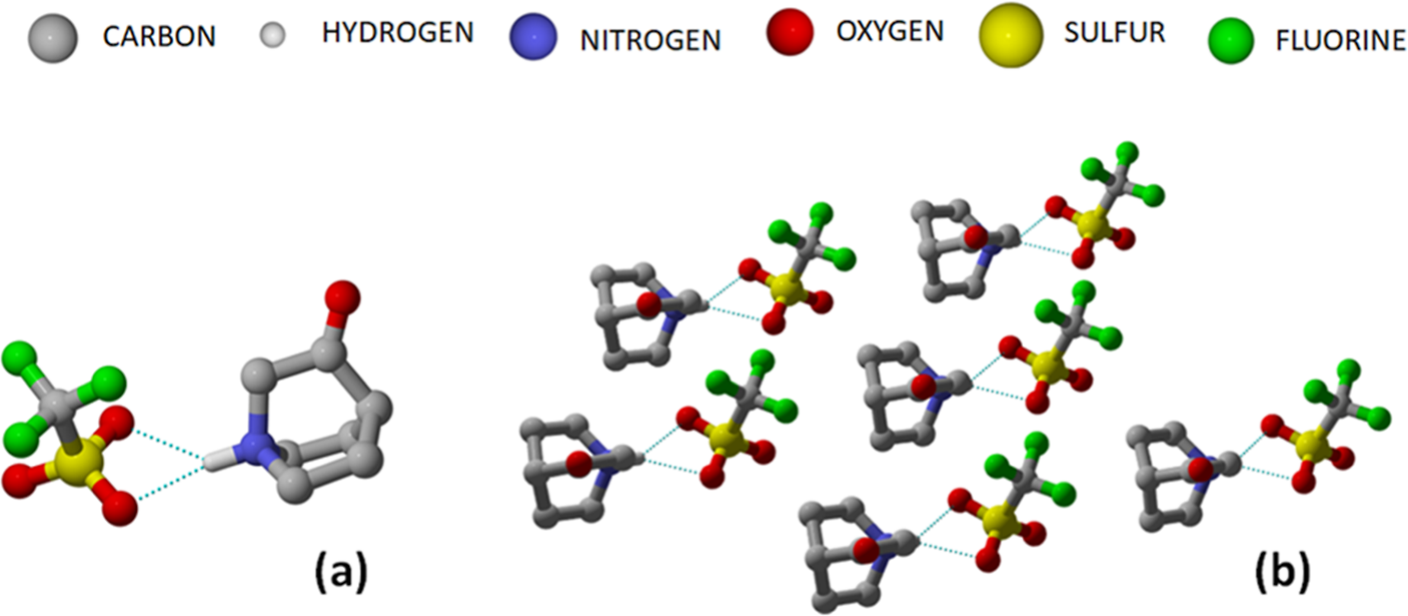
(a) Asymmetric unit of the [QHco]­TFO crystal structure and (b) crystal packing highlighting the bifurcated hydrogen bonds interactions leading to 0D assemblies. H_CH_ atoms are omitted for clarity.

Experimental powder XRD pattern, recorded at RT, matches well with the simulated one from the single crystal structure, revealing the high crystallinity and purity of the phase (Figure SI2).

Conversely, single-crystal analysis at RT on the enantiopure and racemic crystals of R-[QH]­TFO and S/R-[QH]­TFO revealed the same cubic unit cell (*a* = 6.7 Å, *V* = 301 Å^3^), with the *Pm*3̅*m* identified as the most plausible space group, this result is consistent with other reported plastic crystals,^50–52^ as well as with similar systems previously studied by us, based on the R-[QH]^+^ cation paired with PF_6_^–^, BF_4_^–^, CH_3_SO_3_^–^, and halide anions.^7,34,53^ However, the pronounced dynamic disorder affecting both ions in the high-symmetry cubic phase prevented reliable structure solution and refinement.

At the same time, the powder XRD patterns recorded at RT (Figure SI3) show a low number of peaks that reflect a high crystal symmetry, in fact, the same cubic unit cell could be correctly refined via the Pawley method (Figure SI5).

Thermogravimetric analysis (TGA) reveals that all compounds exhibit thermal stability up to approximately 300 °C (Figure SI8). Differential scanning calorimetry (DSC) measurements (Figure SI9), conducted within the temperature range of −50 to 250 °C, detected an endothermic peak at 168.5 °C (Δ*H* = 20.66 J mol^–1^; Δ*S* = 46.80 J mol^–1^) for [QHco]­TFO followed by an exothermic one at 127.3 °C (Δ*H* = 18.55 kJ mol^–1^; Δ*S* = 46.34 J mol^–1^) on cooling, likely corresponding to melting and recrystallization. Compound R-[QH]­TFO showed an exothermic peak at 1.2 °C (Δ*H* = 10.30 kJ mol^–1^; Δ*S* = 37.56 J mol^–1^) on cooling and an endothermic peak at 10.3 °C (Δ*H* = 10.91 kJ mol^–1^; Δ*S* = 38.51 J mol^–1^) on heating, indicating thus a reversible transition. Conversely, the salt S/R-[QH]­TFO showed no detectable peaks within the scanned except a weak endothermic peak at 184 °C (Δ*H* = 0.79 kJ mol^–1^; Δ*S* = 1.73 J mol^–1^) on heating followed by an additional exothermic peak at 167 °C on cooling (Δ*H* = 0.63 kJ mol^–1^; Δ*S* = 1.41 J mol^–1^), likely due to the melting process.

Structural transformations associated with these phenomena were also monitored through Hot Stage Microscopy (HSM) and variable-temperature powder XRD (VT-PXRD). Both techniques were employed to investigate any possible phase transition.

HSM analysis conducted on single-crystal specimens is consistent with DSC results. At RT, only the achiral salt [QHco]­TFO exhibits the characteristic birefringence of ordered crystalline materials in low-symmetry systems such as orthorhombic, monoclinic, or triclinic. This birefringence persists during cooling and, of course, starts to disappear during melting at ca. 170 °C (Figure 4a).

**Figure 4:**
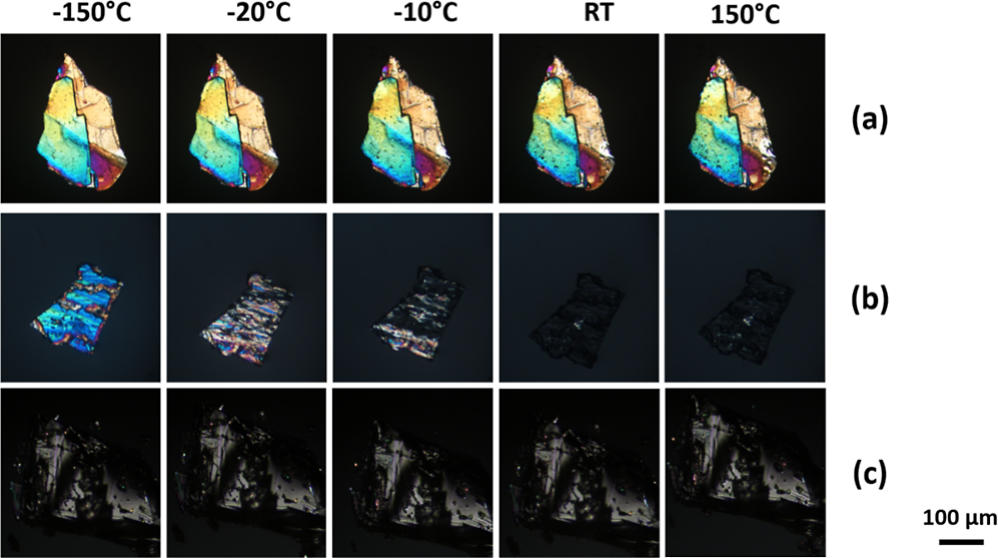
Cross-polarized HSM images recorded at various temperatures for single-crystal samples of (a) [QHco]­TFO, (b) R-[QH]­TFO, and (c) S/R-[QH]­TFO.

On the contrary, both the enantiopure and racemic salts R-[QH]­TFO and S/R-[QH]­TFO do not show any birefringence at RT under cross polarizers. This suggests the presence of a highly symmetric phase, which was subsequently identified as cubic by single-crystal and powder XRD analyses (vide infra). Such highly symmetric phases are commonly associated with plastic crystals, which frequently adopt cubic or hexagonal lattices,^50–52^ and, as we shall see below, the assignment of a plastic phase is further supported by variable-temperature micro Raman measurements, consistent with the presence of dynamic orientational disorder. Interestingly, upon cooling, a neat phase change is observed only for the salt R-[QH]­TFO, as evidenced by the onset of birefringence between −10 °C and −20 °C, indicating that a lower-symmetry, and likely ordered or semiordered phase has been obtained. Additionally, the retention of the crystal’s outer shape, as well as the reversible birefringence loss visible on heating, suggests that such a transformation likely occurs in a single-crystal to single-crystal (SCSC) fashion^54^ (Figure 4b), hypothesis further confirmed by the ϕ-scans XRD patterns at both RT and 250 K (see Figure SI7). Contrary to what expected, for S/R-[QH]­TFO, no birefringence changes were detected on cooling (Figure 4c).

To elucidate the structure of R-[QH]­TFO, an additional data collection at 250 K was performed. At low temperature, the enantiopure salt crystallizes in the orthorhombic system and with the *P*2_1_2_1_2_1_ space group (see Table SI1b for details). Within crystalline R-[QH]­TFO, the TFO^–^ anion features crystallographic disorder that was modeled over two positions with occupancy ratio of 60/40; therefore, this phase can be considered as a semiordered crystal structure. As shown in Figure 5, ionic building blocks establish charge-assisted hydrogen-bonds between cations [N–H···O = 3.089(7) Å] and between cations and anions [N–H···O = 3.11(1)–3.116(9) Å; O–H···O = 2.89(1)–3.12(1) Å].

**Figure 5:**
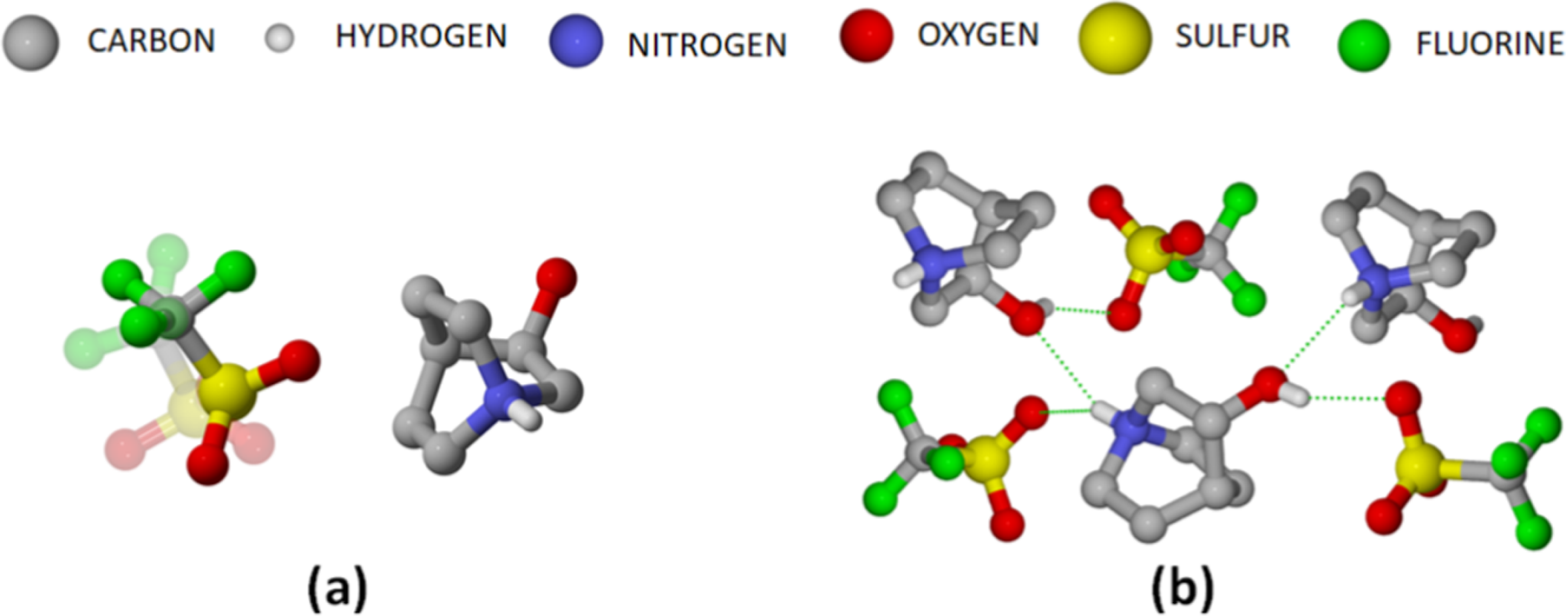
(a) Asymmetric unit of the R-[QH]­TFO crystal structure, showing the disordered TFO^–^ anion with its minor occupancy position represented in transparency, and (b) hydrogen bond pattern leading to 1D tapes. H_CH_ atoms and disorder in (b) omitted for clarity.

VT powder XRD measurements on polycrystalline samples closely align with the DSC and HSM analyses. Upon cooling to the lowest accessible temperature (−150 °C), the achiral salt [QHco]­TFO exhibits no phase transition. However, during heating, a distinct melting event starts around 170 °C (see Figure 6a).

**Figure 6:**
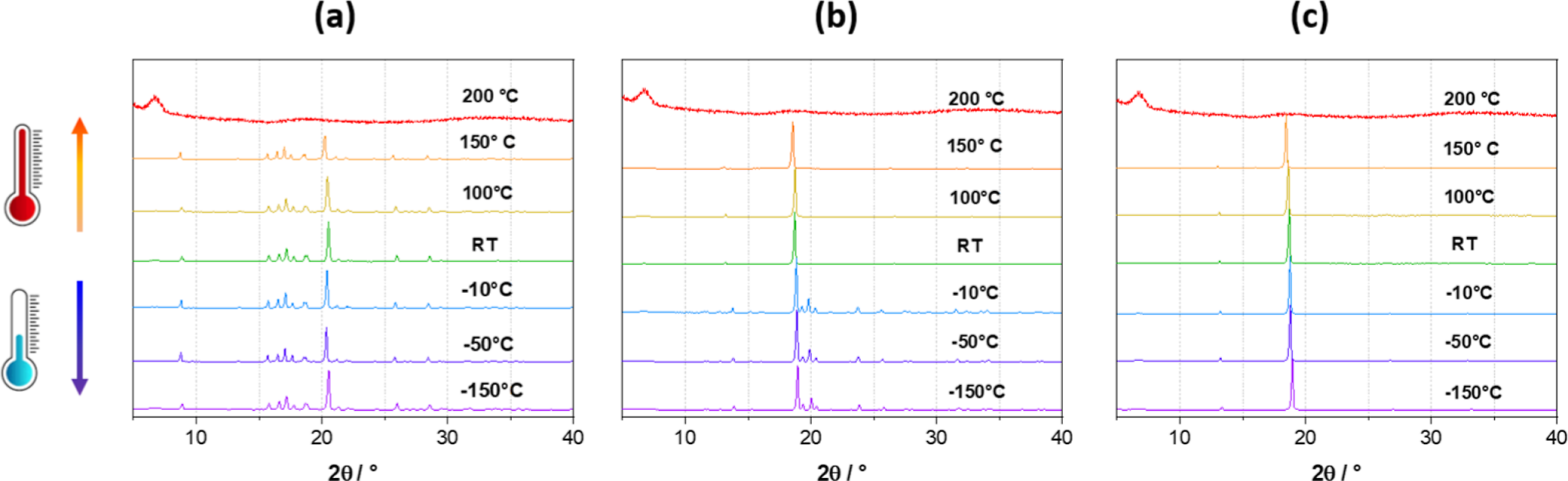
VT powder XRD patterns recorded at various temperatures for polycrystalline samples of: (a) [QHco]­TFO, (b) R-[QH]­TFO, and (c) S/R-[QH]­TFO.

Notably, the enantiopure salt R-[QH]­TFO undergoes a well-defined phase transition between 2 °C and −10 °C. This transition is fully reversible, with the initial phase restored upon heating (Figure 6b), and no other transitions are detected on heating up to the melting.

In contrast, the racemic salt S/R-[QH]­TFO does not exhibit a distinct phase change upon cooling (−150 °C) or during heating except the melting process (Figure 6c).

This is particularly evident when comparing the packings and the relative arrangements of cations and anions found in [QHco]­TFO and R-[QH]­TFO (Figure 7). For [QHco]­TFO, with six anions surrounding each cation, the ionic arrangement is far from a cubic structure, preventing the ordered crystal from rearranging into a plastic phase as the temperature increases. In contrast, in semiordered R-[QH]­TFO, the anions are almost perfectly aligned around the cation, allowing, with minimal motion, the achievement of plastic phase in a cubic system.

**Figure 7:**
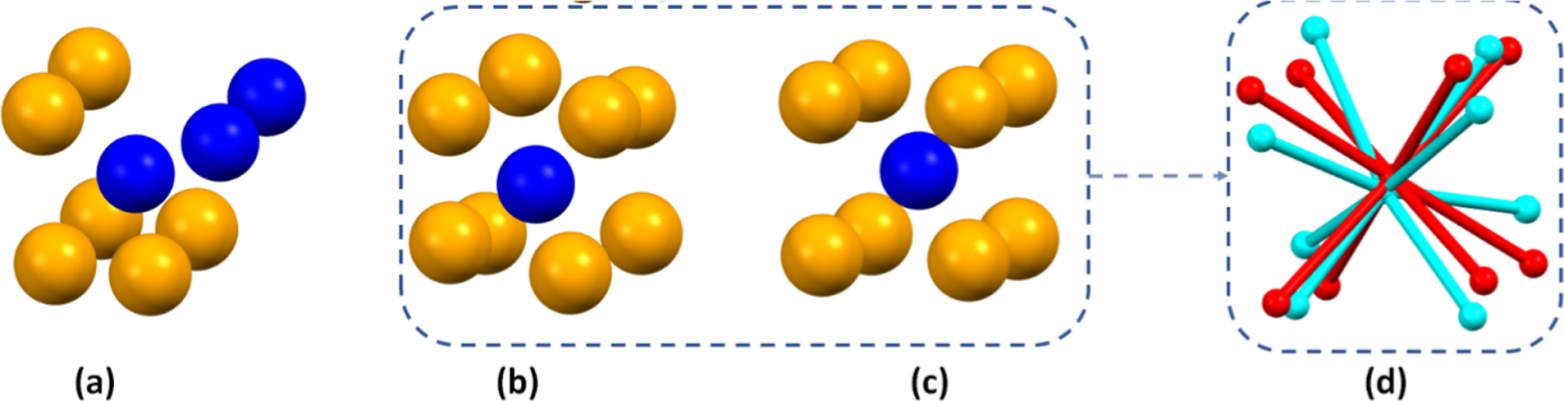
Comparison of ionic environments, with cations and anions are represented by blue and orange spheres, respectively, for (a) [QHco]­TFO, (b) enantiopure R-[QH]­TFO in the semiordered phase, and (c) the cubic plastic phase of R-[QH]­TFO, as simulated based on the determined unit cell and space group. (d) Overlay diagram showing the ionic environment of R-[QH]­TFO in both the semiordered phase (cyan) and plastic phase (red). This overlay highlights the differences between the “quasi-cubic” arrangement and an ideal cubic structure for R-[QH]­TFO.

Studies have emphasized that molecular packing and interlocking are crucial in forming plastic crystals, as they influence steric hindrance and interactions between molecular components,^55,56^ and in regard of this, we have determined the coefficients of linear thermal expansion for [QHco]­TFO and R-[QH]­TFO (see Table 1, Figures SI10 and SI11).

The enantiopure compound R-[QH]­TFO, which undergoes a plastic phase transition, exhibits positive thermal expansion coefficients along all crystallographic axes. In contrast, [QHco]­TFO displays negative thermal expansion along the *a*-axis and no plastic phase transition. Consequently, for [QHco]­TFO, the negative thermal expansion along the *a*-axis may impede molecular reorientation by increasing interlocking and steric hindrance, thus preventing the material from transitioning into a plastic phase with molecular components orientationally disordered.

Following microcalorimetric and VT-XRD analyses, VT Raman spectra were acquired to further characterize the systems and investigate their phase transitions. The low-frequency region (<250 cm^–1^) was used to probe collective lattice vibrations sensitive to intermolecular interactions and crystal packing, while the high-frequency region was used to monitor intramolecular ion modes sensitive to symmetry changes and variations in static or dynamic disorder. This approach, validated in previous investigations of both neutral^57^ and ionic^7,53,57^ plastic crystals, enables the detection of even subtle structural transformations.

In the high-wavenumber region, all samples exhibit the characteristic bands of the TFO^–^ anion, with peak positions varying with crystal composition, symmetry, phase, and disorder. The fingerprint region typically includes a strong S=O stretching band (1030–1045 cm^–1^) and CF_3_ rocking modes (750–770 cm^–1^), often coupled with C–S stretching. These features are sensitive probes of the local anion environment.^58^

The assignments at lower frequencies are more ambiguous and change among the sources, likely due to the mixing of SO_3_, CF_3_ and C–S motions.^59,60^ The internal vibration bands in the studied crystals, have been assigned according to literature retrieved information.^59,60^ Accordingly, the C–S stretching mode appears near 320 cm^–1^, while SO_3_ rocking (ρSO) modes, also involving twisting of CF_3_ and SO_3_ groups, occur around 350 cm^–1^. The asymmetric deformation of the CF_3_ group (δ_as_(CF_3_)) is found near 580 cm^–1^, the asymmetric deformation of SO_3_ (δ_as_(SO_3_)) around 520 cm^–1^, and the symmetric deformation of SO_3_ (δ_s_(SO_3_) between 620 and 625 cm^–1^. The vibrational features of the [QHco]^+^ cation are consistent with those of its protonated analogue [QH]^+^. The symmetric CNC/CCC stretching (ν_s) appears at 807 cm^–1^ in [QHco]^+^ (vs 790 cm^–1^ in [QH]^+^), and the antisymmetric stretching ν_as_(CNC/CCC) is found around 1040 cm^–1^.

The Raman spectrum of the [QHco]­TFO sample is shown in Figure SI13 over the temperature range from 0 to −190 °C and in the spectral range from 10 to 1200 cm^–1^, with the vibrational assignments. In the low-frequency region, the lattice phonon pattern is visible at all temperatures. The overall spectral profile remains essentially unchanged over the entire temperature range, notwithstanding a progressive peak sharpening and improved resolution upon cooling. This confirms the crystalline nature of the system throughout and the absence of any phase transition. The enantiopure R-[QH]­TFO sample exhibits feature typical of plastic crystals, as revealed by its variable-temperature (VT) Raman spectrum shown in Figure 8. At room temperature the low-frequency region is dominated by a broad Rayleigh wing (RW) centered at ω = 0 cm^–1^. This feature is the marker of the dynamic orientational disorder involving both cations and anions in the crystal unit cell. RW is in fact the low-frequency tail of the Rayleigh scattering, arising from quasi-elastic processes linked to slow, large-scale fluctuations such as orientation changes in the medium. As the average cubic structure of the plastic phase lacks well-defined molecular orientations, the Raman lattice phonon pattern is suppressed. A broad band centered at 240 cm^–1^ accompanies the RW and is likely a boson-like signature of collective low-frequency relaxational modes intrinsic to the orientationally disordered plastic crystalline state. This band should not correspond to internal molecular vibrations but rather arise from the presence of quasi-localized modes and dynamic heterogeneity. Upon cooling to −25 °C, the RW disappears, indicating the freezing of dynamic reorientational processes and the formation of a more ordered crystal phase, as confirmed by the appearance of a weakly defined lattice phonon pattern at low frequencies. As the temperature further decreases down to −190 °C, the overall broad low-frequency range gradually gets resolved into sharper phonon-like features. Although this evolution resembles the narrowing of a Boson-like pattern, it most probably reflects the increasing definition of vibrational normal modes allowed by an increased lattice order. Further insights from the internal vibrational energy range of R-[QH]­TFO support this interpretation, with behavior in agreement with the XRD findings of a semiordered phase at low temperature. At RT, the two characteristic bands, i.e. the CF_3_ rocking mode of TFO^–^ at 765 cm^–1^ and the νCNC/CCC stretching mode of [QH]^+^ at 790 cm^–1^, appear fully symmetric, reflecting a highly disordered, dynamically averaged phase.

**Figure 8:**
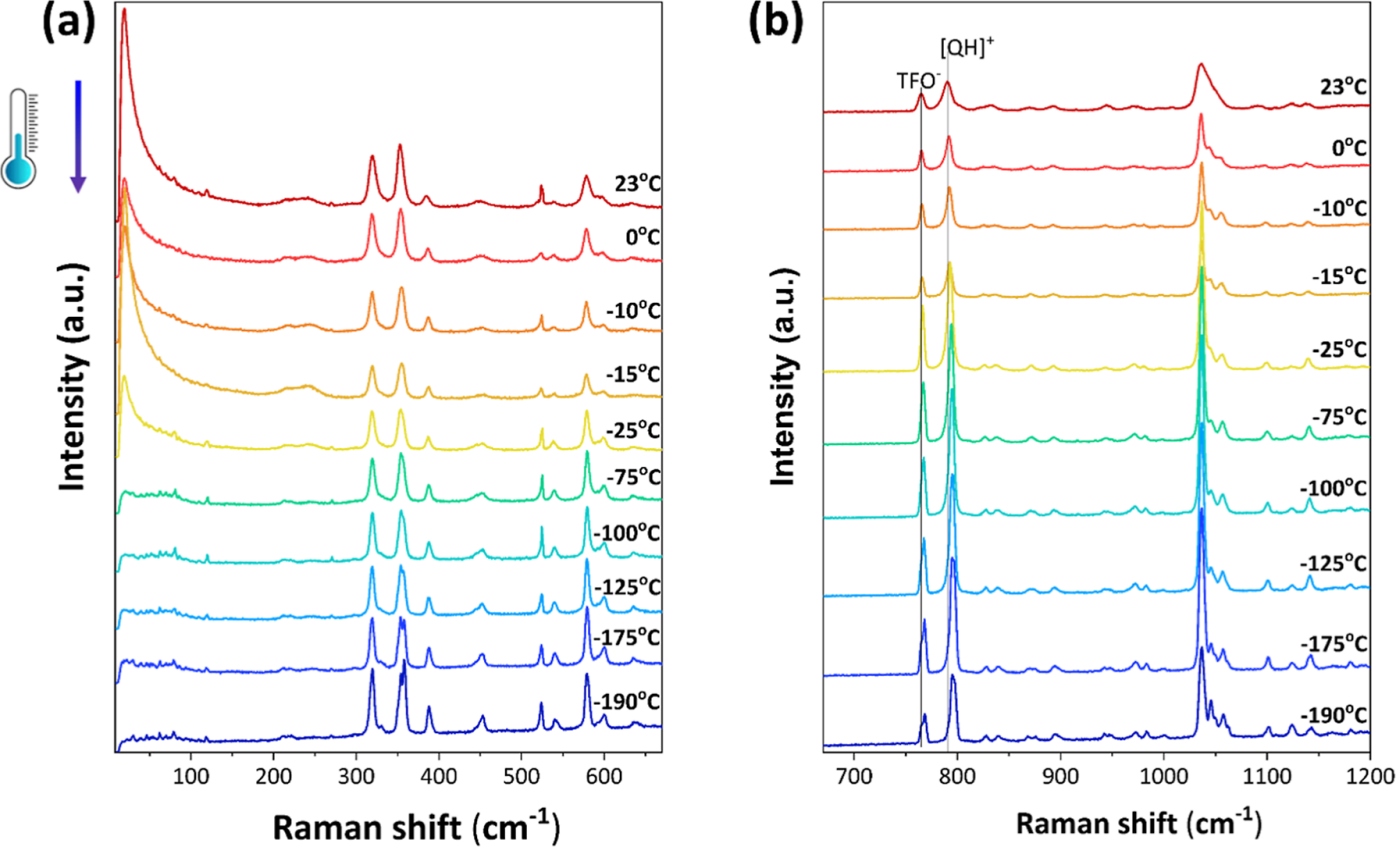
VT Raman spectra for the R-[QH]­TFO as a function of the temperature; (a) lattice phonon region (b) spectral range of the characteristic vibrational modes of the anion TFO^–^ and cation [QH]^+^.

Upon cooling, the CF_3_ rocking band narrows significantly, with its full width at half-maximum (fwhm) decreasing from ∼6.6 cm^–1^ at room temperature to a nearly constant 3.9 ± 0.3 cm^–1^ in the −10 to −50 °C range, while remaining symmetric and essentially unshifted. At −75 °C, however, the band begins to split, as a shoulder emerges on the low-wavenumber side and grows steadily more intense upon further cooling. Although the phase transition to the more ordered monoclinic phase, as detected by XRD, occurs at −16 °C, the onset of asymmetry in the CF_3_ rocking band is only observed at lower temperatures. This delayed onset is likely due to residual dynamic disorder of the anion beyond the phase transition, gradually freezing into static disorder at lower temperatures. This interpretation is consistent with the XRD data, which reveal that the TFO^–^ anion remains disordered over two positions. The skeletal mode band of the cation at ∼790 cm^–1^, very broad in the plastic phase (fwhm of ∼8.5 cm^–1^ at RT) becomes asymmetric around the phase transition, and splits into two narrower components. These can be identified by deconvolution: one with a fwhm of 5.2 ± 0.4 cm^–1^, and the other 4.7 ± 1.1 cm^–1^ in the −10 to −75 °C range. Both components shift progressively to higher wavenumbers on cooling, while maintaining a nearly constant intensity ratio. This behavior suggests that a certain degree of static disorder of the cation is established after the transition, with the progressive shift to higher wavenumbers which is consistent with a progressive stiffening of the local potential as rotational or orientational degrees of freedom become increasingly hindered upon cooling. At −150 °C, the band changes shape again, and a third component becomes detectable. While tentative, this additional feature may suggest a more effective proton sharing between the cation and the anion, possibly driven by a reduced distance between them upon cooling. Although speculative, such a picture would agree with a tightening of the hydrogen-bonding network.

In agreement with the XRD results, the variable-temperature Raman spectra of the racemic S/R-[QH]­TFO (Figure SI14) show no evidence of a phase transition from a dynamically disordered phase to an ordered phase. At room temperature, the spectrum is consistent with a plastic crystal phase, as indicated by the broad RW and the overall broad bands. Upon cooling, a progressive decrease in the RW intensity is observed, and it eventually vanishes at low temperature. However, this suppression of the RW is not accompanied by any appreciable narrowing or splitting of the characteristic intramolecular vibrational modes at 766 cm^–1^ (TFO^–^) and 790 cm^–1^ ([QH]^+^), which remain essentially unchanged. Conversely, on heating from RT up to 150 °C, the spectrum shows a marked increase in RW intensity and the persistence of broad bands, confirming the typical vibrational signature of a plastic crystalline state and increased dynamic disorder at high temperature.

As noted above, the finding that the racemic salt S/R-[QH]­TFO did not undergo any transition to an ordered or semiordered phase upon cooling was unexpected. This behavior could be interpreted as a direct consequence of the different stereochemistry of the R-[QH]^+^ and S-[QH]^+^ cations, the fact that crystallization takes place above the plastic phase transition temperature, therefore both enantiomers can occupy indistinct crystallographic positions, and ion mobility, which is not enough to allow complete cation migration within the crystal lattice.

These factors prevent the system from reorganizing into either separated enantiopure phases or an ordered racemate^61–63^ Instead, the material remains permanently disordered.

This behavior can be rationalized in terms of substitutional disorder. In the plastic phase, shape similarity allows R- and S-[QH]^+^ cations to occupy equivalent crystallographic sites; however, their statistical distribution hinders the cooperative reorganization required to form a long-range ordered phase upon cooling, as depicted in Scheme 2. The resulting configurational entropy, together with local packing constraints and limited ion mobility, likely contributes to the stabilization of the disordered state. In this sense, the present system represents an enantiomeric analogue of the “reordering frustration” previously reported for anion-substituted ionic plastic crystal solid solutions,^53^ and to the best of these authors’ knowledge, this system represents the second known example of a crystalline material with a relatively simple composition displaying such behavior.

**Scheme 2:**
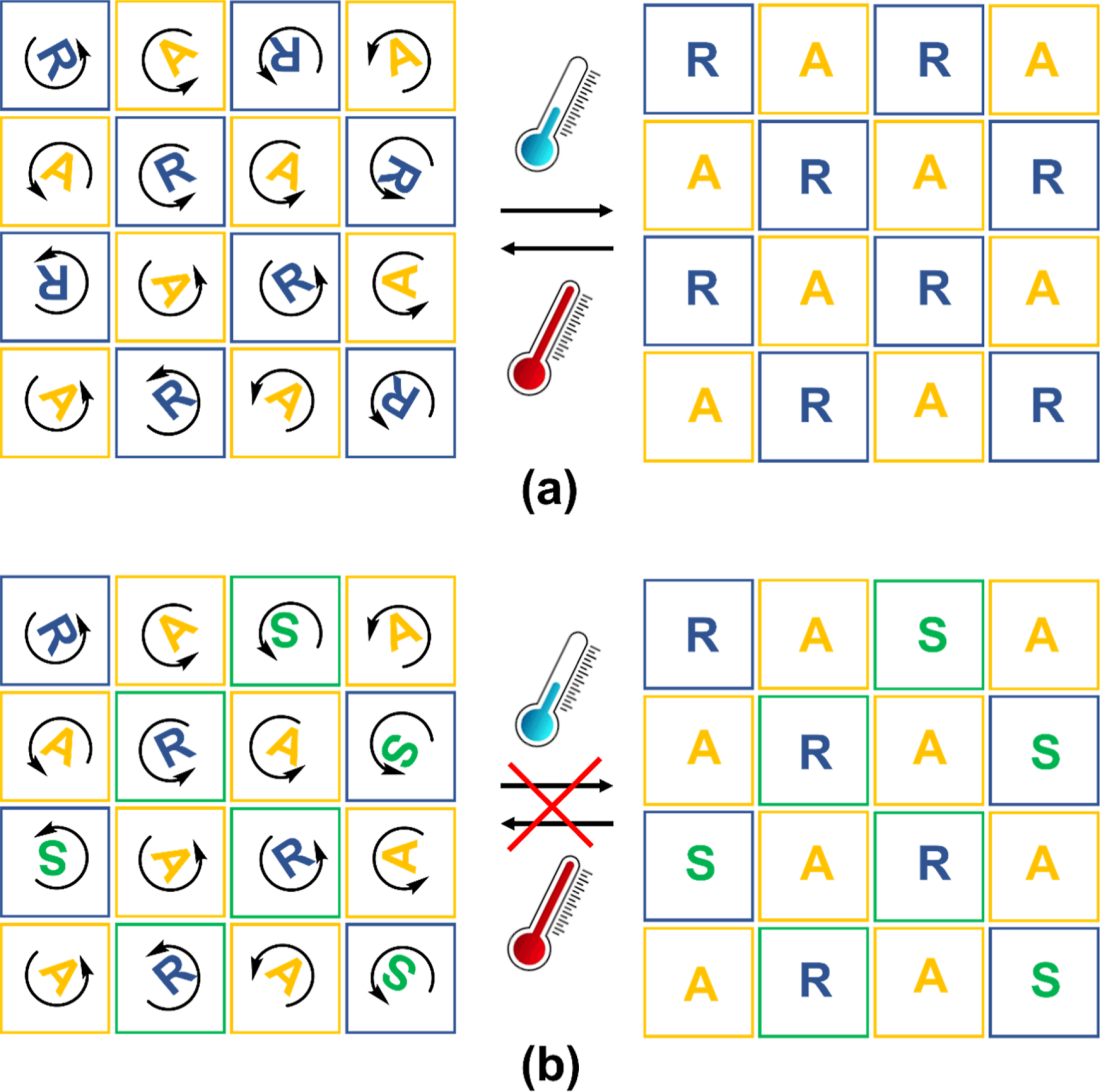
(a) Representation of the Reordering Transition from Plastic-to-Ordered Phase in Crystalline R-[QH]­TFO and (b) Depiction of “Reordering Frustration” in Crystalline S/R-[QH]­TFO^a^

a Blue and green squares mark the crystallographic positions of the R-[QH]^+^ and S-[QH]^+^ cations, represented as R (blue) and S (green). Orange squares aignify the crystallographic site of the TFO^–^ anion, depicted as a (orange). Black arrows represent dynamic motion affecting the ionic building blocks in plastic phase.

To test this hypothesis, crystallization experiments were also performed below the presumed transition temperature. However, all crystals still displayed the same cubic unit cell both at RT and 100 K, with no evidence of phase transition.

Therefore, we focused on preparing solid solutions of R-[QH]­TFO and S/R-[QH]­TFO by mixing them in the following ratios: 25/75, 50/50, and 75/25. The resulting crystalline phases correspond to compositions of R-[QH]_*x*_·S-[QH]_(1–*x*)_TFO, with *x* values of 0.63, 0.75, and 0.87. We then analyzed their structural properties and thermal behavior.

Notably, this process involves enriching the crystal lattice of the racemic salt with the R-enantiomer. If our assumption holds—that in S/R-[QH]­TFO, the R-[QH]^+^ and S-[QH]^+^ cations are indistinguishable due to their crystallographic within the plastic phase—then the resulting structures should exhibit permanent disorder even at low temperatures. As expected, the solid solutions R-[QH]_*x*_·S-[QH]_(1–*x*)_TFO (*x* = 0.63, 0.75, and 0.87) are isomorphous among them and do not display any phase transition to ordered or semiordered structures on lowering the temperature as witnessed by VT powder XRD and HSM analyses (Figure 9). Additionally, solid-solution presents an increased melting temperature, as witnessed by the powder XRD pattern recorded at 200 °C, where the R-[QH_]0.75_·S-[QH]_0.25_TFO phase is not melted, unlike its parent compounds.

**Figure 9:**
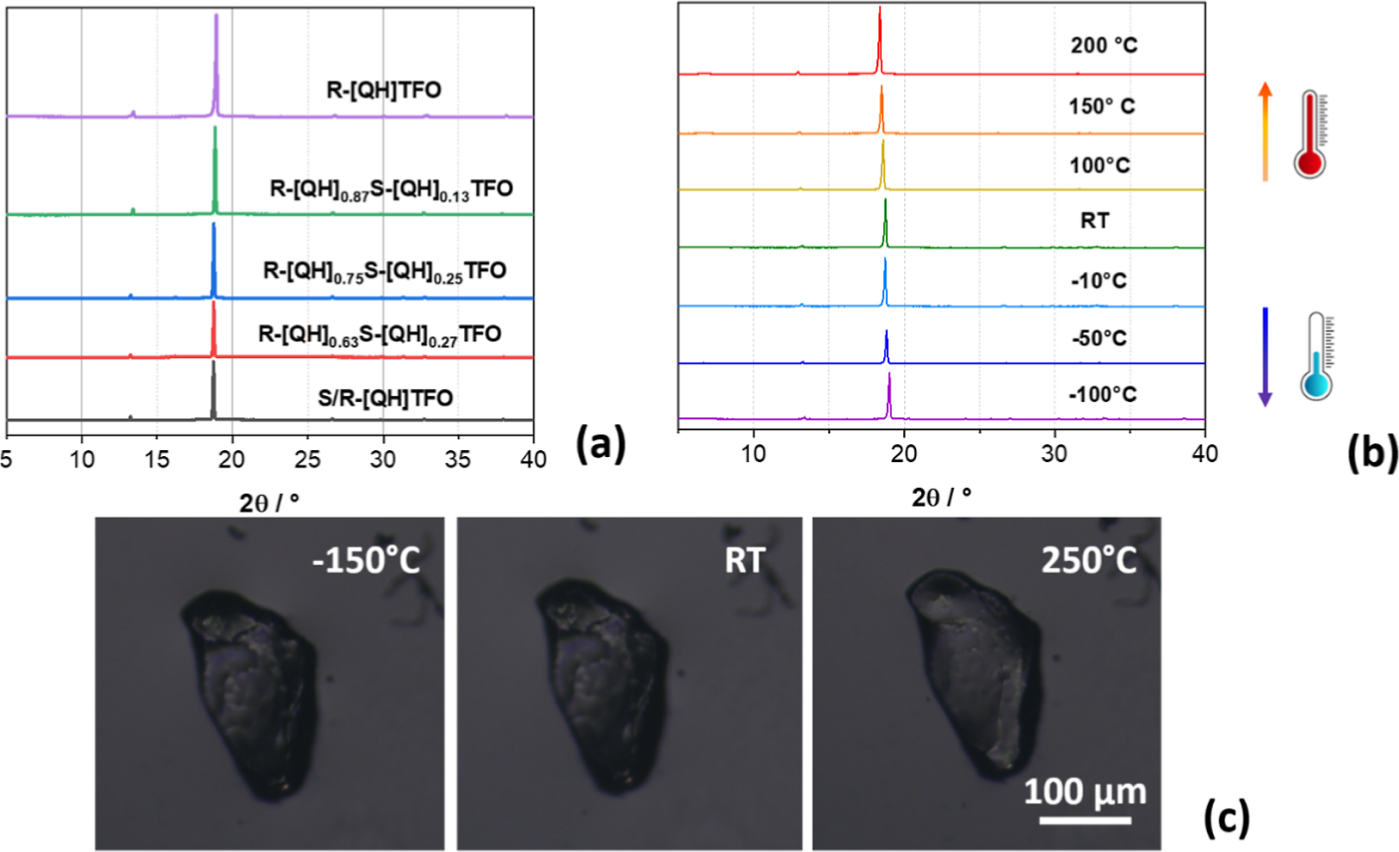
(a) Powder XRD patterns of R-[QH]_*x*_·S-[QH]_(1–*x*)_TFO (*x* = 0.63, 0.75, and 0.87) solid-solutions, (b) VT powder XRD patterns for R-[QH]_0.75_·S-[QH]_0.25_TFO, and (c) cross-polarized HSM images of a single crystal of R-[QH]_0.75_·S-[QH]_0.25_TFO. No phase transitions or birefringence changes were observed upon cooling.

It is important to stress that this system exhibits a complete lack of enantioselectivity from a crystallographic perspective.^64,65^ The structural basis for such solid solution formation is as follows: nonracemic mixtures develop quasi-centrosymmetric structures where the quantity of the absent enantiomer is offset by the more abundant one adopting a conformation that resembles the minority enantiomer.^66^ This phenomenon is known as shape mimicry.^67^ Consequently, the enantiopure and racemic structures are isomorphic, allowing for a continuous change in enantiomeric composition while maintaining the same crystal packing.

On the other hand, and as expected for crystalline species that do not share the same capability to establish similar hydrogen-bond interaction patterns, attempts to form solid solutions between the pairs S/R-[QH]­TFO/[QHco]­TFO and R-[QH]­TFO/[QHco]­TFO were unsuccessful and resulted only in physical mixtures, as indicated by the powder XRD patterns in Figure SI6.

Complementary VT Raman spectroscopic analyses (see Figure 10) further support the absence of any transition to a semiordered or fully ordered phase in the solid solution R-[QH]_*x*_·S-[QH]_1–*x*_·[TFO] (*x* = 0.75). Consistent with variable-temperature XRD and microcalorimetric findings, no clear spectral signatures of long-range ordering are observed across the entire investigated temperature range. Upon cooling from RT to below −50 °C, the characteristic intramolecular vibrational modes of TFO^–^ (766 cm^–1^) and [QH]^+^ (790 cm^–1^) exhibit sharpening and a slight increase in intensity, accompanied by a progressive decrease in the Rayleigh wing, indicating a gradual reduction in dynamic disorder and the formation of a statistically populated, statically frozen disordered state, i.e., the glassy crystalline phase. Conversely, upon heating to 150 °C, the RW grows markedly in both intensity and sharpness, consistent with enhanced dynamic motions (Figure 10). The temperature-dependent changes observed in this spectral region, including the suppression of the sharp feature around 520 cm^–1^ assigned to an internal SO_3_ deformation mode, are not associated with a long-range phase transition, as no corresponding VT-XRD, HSM or DSC event is observed. They are more reasonably attributed to the sensitivity of this TFO^–^ vibration to the local dynamical environment of the anion, involving motional averaging, Raman tensor polarization effects and possible temperature-induced broadening.

**Figure 10:**
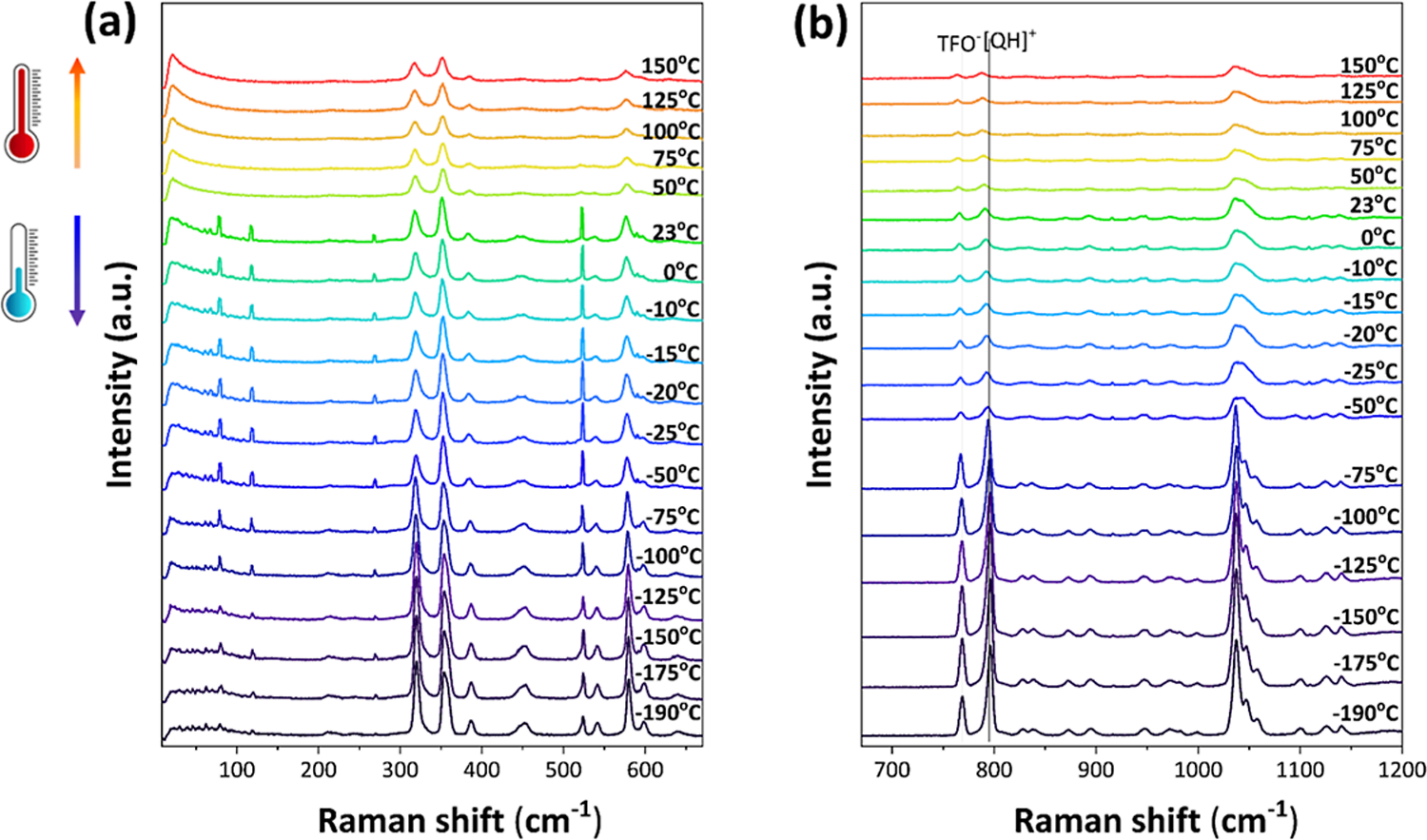
VT Raman spectra for the solid solution R-[QH]_*x*_·S-[QH]_1–*x*_·[TFO] (*x* = 0.75) as a function of the temperature; (a) lattice phonon region (b) spectral range of the characteristic vibrational modes of the anion TFO^–^ and cation [QH]^+^ for the solid solution.

These results highlight the specific role of low-frequency Raman spectroscopy in distinguishing dynamically disordered, statically disordered, and progressively ordered states. While XRD defines the average crystallographic structure, the Rayleigh wing and the evolution of the lattice-phonon pattern reveal whether orientational disorder remains dynamic or becomes progressively frozen upon cooling. This distinction is essential for understanding the functional behavior of these compounds, since proton transport is expected to depend not only on the available structural pathways, but also on the dynamical fluctuations that create, maintain, or hinder their connectivity. The present results therefore indicate that the conventional structure–property picture should be expanded to explicitly include lattice dynamics.

Finally, the proton conductivity of [QHco]­TFO, S/R-[QH]­TFO, R-[QH]­TFO, and solid solution R-[QH]_0.75_·S-[QH]_0.25_·[TFO] was investigated by EIS within their thermally stable ranges and under anhydrous conditions. Pellets of the samples were placed between two stainless steel blocking electrodes housed in a two-electrode cell and tested in a dry room (see Experimental Section). It is worth noting that, the plastic phases yielded pellets that were more mechanically stable and reproducible, consistent with their lower stiffness and enhanced deformability, as generally observed for plastic crystalline materials.^14^

The Nyquist plots of the investigated samples are reported in Figure SI15. The ac-spectra were acquired from 30 °C up to 140 °C with 10 °C intervals. All the impedance spectra are characterized by an arc whose magnitude decreases with the increase of the set temperature. This feature is addressed to the migration of ions through the electrolyte, coupled with the dielectric capacitance of the cell setup.

In the case of R-[QH]­TFO, S/R­[QH]­TFO, and R­[OH]_0.75_-S­[OH]_0.25_TFO, a linear decrease of the overall impedance is observed with the increase of temperature. On the other hand, in the case of [QHco]­TFO, the overall impedance from 30 °C up to 80 °C was out of the instrument capability. From 90 °C up to 140 °C, the material has shown conductive behavior with a steep decrease of overall impedance as the temperature increased, suggesting a possible high activation energy for the conduction mechanism.

The impedance spectrum was fitted with an (RQ)­Q ECM, i.e., a resistor in parallel with a constant phase element, to account for the resistance associated with the ionic conductivity of the electrolyte and the dielectric capacitance of the cell setup, respectively. The ionic conductivity σ (S cm^–1^) was then calculated according to eq 1 ^68^

1 $$\sigma =\frac{l}{RA}$$

In which *R* is the calculated resistance from the fit procedure, *l* is the thickness (in cm) of the electrolyte pellet, and *A* represents the geometrical area of the pellet (1.33 cm^2^). In both materials, the ionic conductivity linearly increases vs 1000 *T* ^–1^. The activation energies for each electrolyte were calculated according to eq 2 ^69^

2 $$\ln\sigma =\ln\sigma_{0}-\frac{E_{a}}{k_{b}T}$$

where σ_0_ is the pre-exponential factor and graphically corresponds to the intercept of the linear fit, *E*_a_ is the activation energy, *k*_b_ is the Boltzmann’s constant (8.617 × 10^–5^ eV K^–1^), and T the temperature. The activation energy *E*_a_ can be derived from the linear fit of the plots shown in Figure 11. The results are summarized in Table 2.

**Figure 11:**
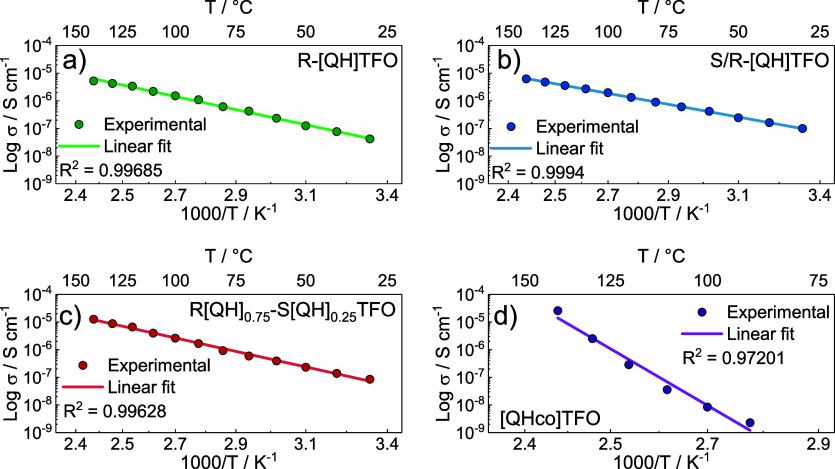
Arrhenius plots at different temperatures: (a) R­[QH]­TFO, (b) S/R­[QH]­TFO, (c) R­[QH]_0.75_-S­[QH]_0.25_TFO, and (d) [QHco]­TFO.

**Table 2:** Summary of the Calculated Ionic Conductivities and Activation Energies of the Compounds [QHco]­TFO, S/R-[QH]­TFO, R-[QH]­TFO, and R­[QH]_0.75_-S [QH]_0.25_TFO

	[QHco]TFO	S/R-[QH]TFO	R-[QH]TFO	R[QH]_0.75_-S[QH]_0.25_TFO
σ/S cm^–1^ (*T* = 30 °C)	2.3 × 10^–9^ (*T* = 90 °C)	9.9 × 10^–8^	4.1 × 10^–8^	8.4 × 10^–8^
σ/S cm^–1^ (*T* = 140 °C)	2.5 × 10^–5^	6.1 × 10^–6^	5.2 × 10^–6^	1.3 × 10^–5^
*E*_a_/kJ mol^–1^	233.4	39.66	47.08	48.63
*E*_a_/eV	2.419	0.411	0.488	0.504
*R* ^2^	0.97201	0.9994	0.99685	0.99628

The enantiopure compound R-[QH]­TFO, the racemic S/R-[QH]­TFO, and the solid solution R-[QH]_0.75_-S-[QH]­TFO_0.25_ have shown a linear relationship between the measured ionic conductivity vs 1000/T K^–1^ (Figure 11a–c), a typical Arrhenius behavior for energy activated processes. The enantiopure R-[QH]­TFO has shown the lowest ionic conductivity and activation energy among the series, with a σ = 4.1 × 10^–8^ S cm^–1^ and σ = 5.2 × 10^–6^ S cm^–1^ at 30 and 140 °C, respectively. When the racemic mixture, S/R-[QH]­TFO, is employed, the highest ionic conductivity at 30 °C among the series was measured. On the other hand, the activation energy has shown a significant decrease (39.66 kJ mol^–1^ vs 47.08 kJ mol^–1^ for S/R-[QH]­TFO and R-[QH]­TFO, respectively), proving a higher probability of successful transport events at low temperatures. In the case of the solid solution R-[QH]_0.75_·S-[QH]_0.25_TFO, an ionic conductivity of 8.4 × 10^–8^ S cm^–1^ at 30 °C was measured, slightly lower than the racemic mixture. In addition, the solid solution has shown the highest activation energy among the hydroxyquinuclidinium, with a value of 48.63 kJ mol^–1^, which led to the highest ionic conductivity measured at *T* = 140 °C i.e., σ = 1.3 × 10^–5^ S cm^–1^, i.e., 1 order of magnitude higher than R-[QH]­TFO and S/R-[QH]­TFO). Given the obtained activation energies (all of them lower than 0.5 eV K^–1^), it can be inferred that the H^+^ conduction in the tested materials is mainly given by Grotthuss mechanism (proton hopping).^10,70^

In summary, at low temperatures, R­[QH]_0.75_-S­[QH]_0.25_TFO displays a lower ionic conductivity, as the larger energy barrier for proton hopping reduces the probability of successful transport events. Indeed, this behavior is consistent with the thermally activated nature of proton conduction by Grotthuss mechanism.^10,70^ However, upon increasing the temperature, the additional thermal energy compensates for the higher activation barrier, and the intrinsic structural features of R­[QH]_0.75_-S [QH]_0.25_TFO become dominant: the possible larger number of available conduction pathways and/or a higher concentration of mobile ions, results in a significantly enhanced conductivity.

In the case of [QHco]­TFO (Figure 11d), a high activation energy was observed (233.4 kJ mol^–1^) which suggests that the proton conduction is strongly impeded at low temperatures. Indeed, the overall impedance of the material at *T* < 90 °C was high enough to be out of the instrument capability. In this case, it may be inferred that the higher degree of order in [QHco]­TFO led to a lower number of available conduction pathways, resulting in a less probable transport event; thus, a lower conductivity at lower temperatures.

## Conclusions

This study demonstrates how subtle variations in cation shape and chirality within a closely related family of triflate salts govern crystal packing, phase transition behavior, solid-solution formation, and ultimately proton-transport properties. Despite their compositional similarity, the three investigated systems exhibit markedly different solid-state behaviors. The achiral [QHco]­TFO crystallizes in a fully ordered phase at room temperature and does not display any transition to a dynamically disordered state. In contrast, both R-[QH]­TFO and S/R-[QH]­TFO adopt orientationally disordered yet translationally ordered phases at room temperature, consistent with the structural features of ionic plastic crystals (IPCs). Upon cooling, only the enantiopure R-[QH]­TFO undergoes a plastic-to-ordered transition, whereas the racemic S/R-[QH]­TFO retains its plastic character down to the lowest investigated temperature.

Solid-solution formation proved highly selective: among the three binary systems examined, only S/R-[QH]­TFO/R-[QH]­TFO yielded genuine solid solutions with general formula: R­[QH]_*x*_-S­[QH]_(1–*x*)_TFO (*x* = 0.63, 0.75, and 0.87), while combinations involving [QHco]­TFO resulted in physical mixtures. Notably, variable temperature powder XRD, microcalorimetric analyses, and Micro-Raman investigations carried out on the R­[QH]_0.75_-S [QH]_0.25_TFO solid solution revealed that this phase stabilizes an orientationally disordered ionic plastic crystal phase over a broad temperature range. This persistence of the plastic state can be rationalized in terms of “reordering frustration,” arising from the statistical distribution of racemic and enantiopure species within the lattice, which suppresses long-range reordering and prevents the symmetry lowering observed in the pure enantiomer.

The functional implications of this frustration-stabilized plastic state are clearly reflected in the transport properties. While the fully ordered [QHco]­TFO is not capable of conducting protons under dry and inert conditions, both R-[QH]­TFO and S/R-[QH]­TFO exhibit comparable proton conductivity at room and high temperatures, with similar activation energies, consistent with analogous thermally activated transport mechanisms operating within their plastic states. Strikingly, the S/R-[QH]­TFO/R-[QH]­TFO solid solution displays a significant enhancement in proton conductivity at high temperature, reaching values approximately 1 order of magnitude higher than those of the parent compounds, while maintaining comparable activation barriers. This indicates that the improved performance does not arise from a change in the fundamental transport mechanism, but rather from an increased density and connectivity of accessible proton-transfer pathways enabled by the extended orientationally disordered plastic phase.

Importantly, the low-frequency Raman results show that the functional behavior of these systems is ruled also by the dynamical state of their orientational disorder. The Rayleigh wing, its temperature dependent suppression, and the recovery of lattice phonon features provide direct evidence that the functional properties are better understood within a structure-dynamics-property framework.

Overall, these results demonstrate that crystal engineering of solid solutions represents an effective tool for optimizing functional properties in molecular ionic solids, where controlled lattice disorder and its dynamical state can be deliberately harnessed to enhance physicochemical properties such as proton transport.

## Supplementary Material



## Notes

Crystal data and refinement details, powder XRD patterns comparison and Pawley refinement plots, ϕ-scan images taken on a single crystal of R-[QH]­TFO at RT and 100 K, thermal analyses (TGA thermograms and DSC traces), relative change in principal axis length for [QHco]­TFO and R-[QH]­TFO, indicatrix plots for [QHco]­TFO and R-[QH]­TFO, Raman spectroscopy, electrochemical impedance spectroscopy (Nyquist plots) (PDF)
