# Supplementary material for: Reordering Frustration in Enantiomer-Enriched Solid Solutions of Ionic Plastic Crystals for Proton-Conducting Materials Design
Source: Chem Mater. 2026 Jun 27;38(13):6781–95. doi: 10.1021/acs.chemmater.6c00948 (PMC13374734; doi:10.1021/acs.chemmater.6c00948)
Supplement: Supplementary file 1 [file cm6c00948_si_001.pdf]

Reordering Frustration in Enantiomer-Enriched Solid Solutions of Ionic Plastic Crystals for Proton-Conducting Materials Design

Andrea Vitale, [a,ǂ] *Wenjing Chen,*[b,ǂ] *Antunes Staffolani,**[*,a,c] *Massimo Marcaccio,*[a] *Elisabetta Venuti,*[b] *Tommaso Salzillo,**[*,b] *and Simone d'Agostino**[*,a]

[a] Department of Chemistry "Giacomo Ciamician", the University of Bologna, Via P. Gobetti 85, 40129, Bologna, Italy.

[b] Department of Industrial Chemistry "Toso Montanari", the University of Bologna, Via P. Gobetti 85, 40129, Bologna, Italy.

[c] ENERCube, Centro Ricerche Energia, Ambiente e Mare, Centro Interdipartimentale per la Ricerca Industriale Fonti Rinnovabili, Ambiente, Mare ed Energia (CIRI-FRAME)—Alma Mater Studiorum University of Bologna, Viale Ciro Menotti, 48, 48122 Marina di Ravenna, Italy

[ǂ] These authors contributed equally to this work.

Supporting Information (11 pages)

Table of contents

Force curve of the quartz support	SI-2
Crystal data and refinement details for [QHco]TFO, S/R-[QH]MS, and R-[QH]MS	SI-3
Powder XRD patterns comparison and Pawley refinement plots	SI-5
φ-Scan images taken on a single crystal of R-[QH]TFO at RT and 100K	SI-7
Thermal Analyses (TGA thermograms and DSC traces)	SI-8
Relative change in principal axis length for [QHco]TFO and R-[QH]TFO	SI-9
Indicatrix plots for [QHco]TFO and R-[QH]TFO	SI-11
Raman spectroscopy	SI-12
Electrochemical Impedance Spectroscopy (Nyquist Plots)	SI-13

Force curve of the quartz support

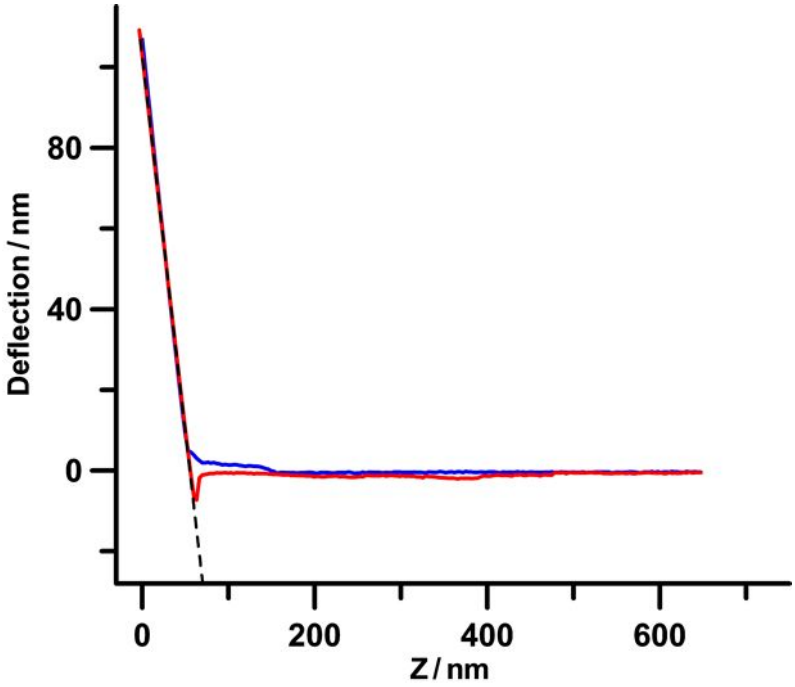

Figure SI1. Force curve, deflection vs tip travel ramp (z-scan) on the quartz glass slide. Blue trace is the approaching curve; red trace is the retracting curve; black dashed straight line is the interpolation of the first points of the retracting curve (red curve).

Crystal data and refinement details

Table SI1a. Crystal data and refinement details for crystalline [QHco]TFO at VT.

	[QHco]TFO				
**Temperature / K**	100	250	200	250	300
**Formula**	$C_8H_{12}F_3NO_4S$	$C_8H_{12}F_3NO_4S$	$C_8H_{12}F_3NO_4S$	$C_8H_{12}F_3NO_4S$	$C_8H_{12}F_3NO_4S$
**FW (g/mol)**	275.25	275.25	275.25	275.25	275.25
**Crystal System**	Monoclinic	Monoclinic	Monoclinic	Monoclinic	Monoclinic
**Space Group**	$P2_1/n$	$P2_1/n$	$P2_1/n$	$P2_1/n$	$P2_1/n$
**a/Å**	8.3174(6)	8.3370(3)	8.3476(3)	8.3722(3)	8.4074(3)
**b/Å**	6.3207(4)	6.3812(3)	6.4267(3)	6.4740(4)	6.5313(2)
**c/Å**	20.4503(10)	20.4475(7)	20.4129(6)	20.4210(7)	20.4235(6)
**α/°**	90	90	90	90	90
**β/°**	92.569(5)	92.514(4)	92.310(3)	92.000(4)	91.644(3)
**γ/°**	90	90	90	90	90
**Volume/Å^3**	1074.03(12)	1086.76(7)	1094.21(7)	1106.18(9)	1121.02(6)
**Z**	4	4	4	4	4
**ρ_{calc} g/cm^3**	1.702	1.682	1.671	1.653	1.631
**μ/mm^{-1}**	0.348	0.344	0.342	0.338	0.334
**measd rflns**	4671	4858	4650	5330	8272
**indep rflns**	2428	2477	2485	2538	2702
**R_1**	0.0467	0.0452	0.0605	0.0586	0.0691
**wR_2**	0.1063	0.1000	0.1898	0.1450	0.2051

Table SI1b. Crystal data and refinement details for crystalline R-[QH]TFO TFO at VT.

	**R-[QH]TFO**			
**Temperature / K**	100	150	200	250
**Formula**	$C_8H_{14}F_3NO_4S$	$C_8H_{14}F_3NO_4S$	$C_8H_{14}F_3NO_4S$	$C_8H_{14}F_3NO_4S$
**FW (g/mol)**	277.26	277.26	277.26	277.26
**Crystal System**	orthorhombic	orthorhombic	orthorhombic	orthorhombic
**Space Group**	$P2_12_12_1$	$P2_12_12_1$	$P2_12_12_1$	$P2_12_12_1$
**a/Å**	8.7330(15)	8.7673(16)	8.8131(9)	8.8847(6)
**b/Å**	9.0977(10)	9.1135(11)	9.1136(6)	9.1361(5)
**c/Å**	13.6940(17)	13.7257(17)	13.7611(10)	13.8031(6)
**α/°**	90	90	90	90
**β/°**	90	90	90	90
**γ/°**	90	90	90	90
**Volume/Å³**	1088.0(3)	1096.7(3)	1105.28(16)	1120.42(11)
**Z**	4	4	4	4
**ρ_{calc} g/cm³**	1.693	1.679	1.666	1.644
**μ/mm⁻¹**	0.344	0.342	0.339	0.334
**measd rflns**	4866	3265	3566	3620
**indep rflns**	2428	2270	2371	2396
**R_1**	0.0950	0.1098	0.0694	0.1279
**wR_2**	0.2448	0.1574	0.1279	0.1780

Powder XRD patterns comparison and Pawley refinement plots

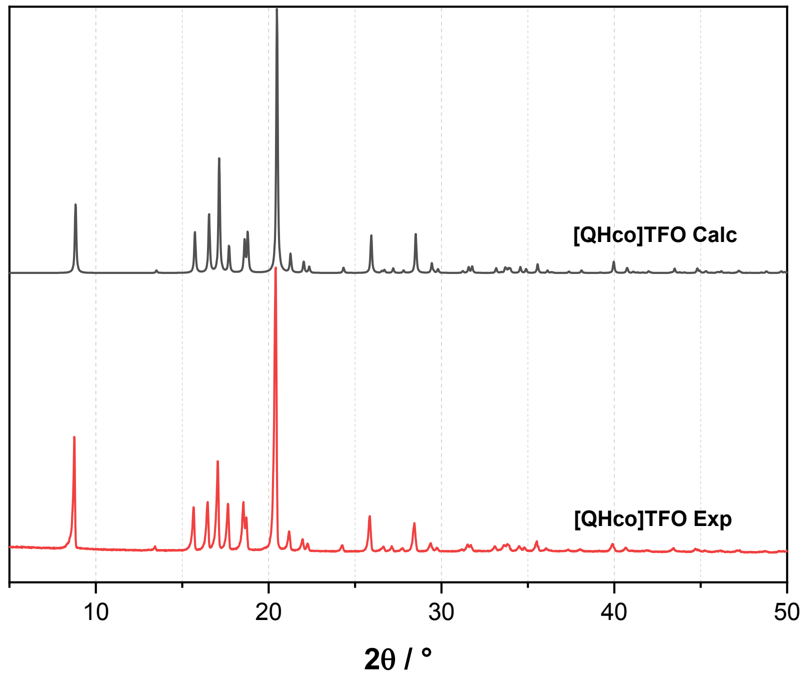

Figure SI2. Comparison between the calculated and experimental powder XRD pattern recorded at RT for a polycrystalline sample of [QHco]TFO.

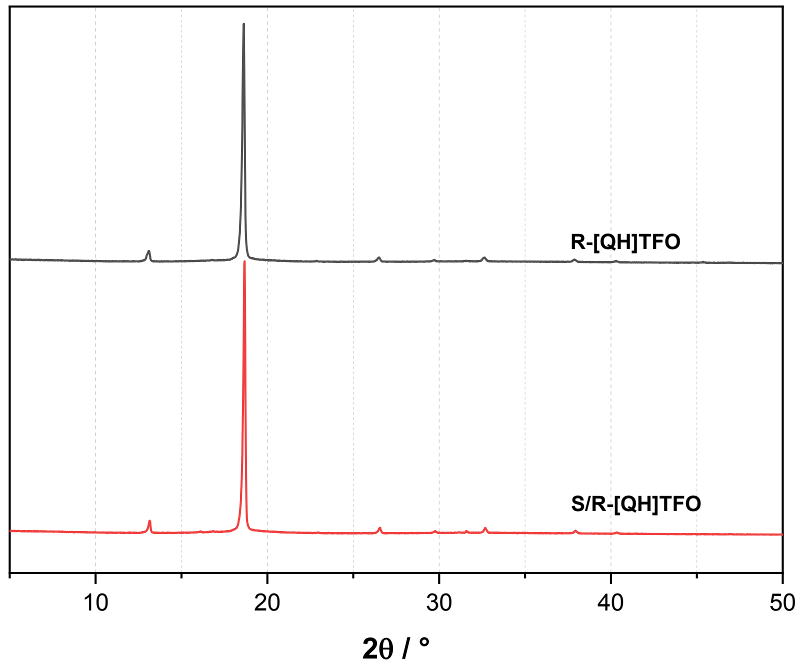

Figure SI3. Experimental powder XRD patterns recorded at RT for (a) R-[QH]TFO and (b) S/R-[QH]TFO.

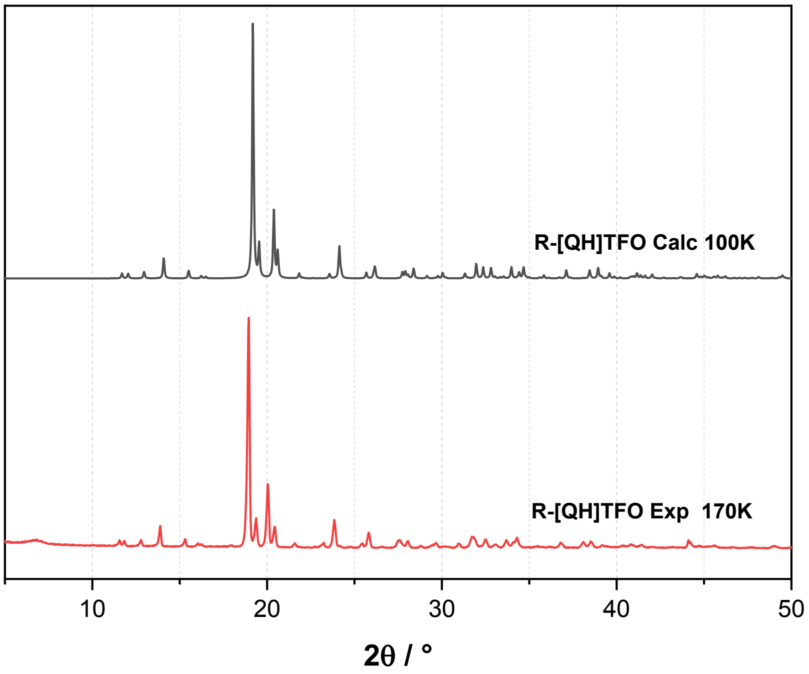

Figure SI4. Comparison between the calculated and experimental powder XRD pattern recorded at 100K for a polycrystalline sample of R-[QH]TFO.

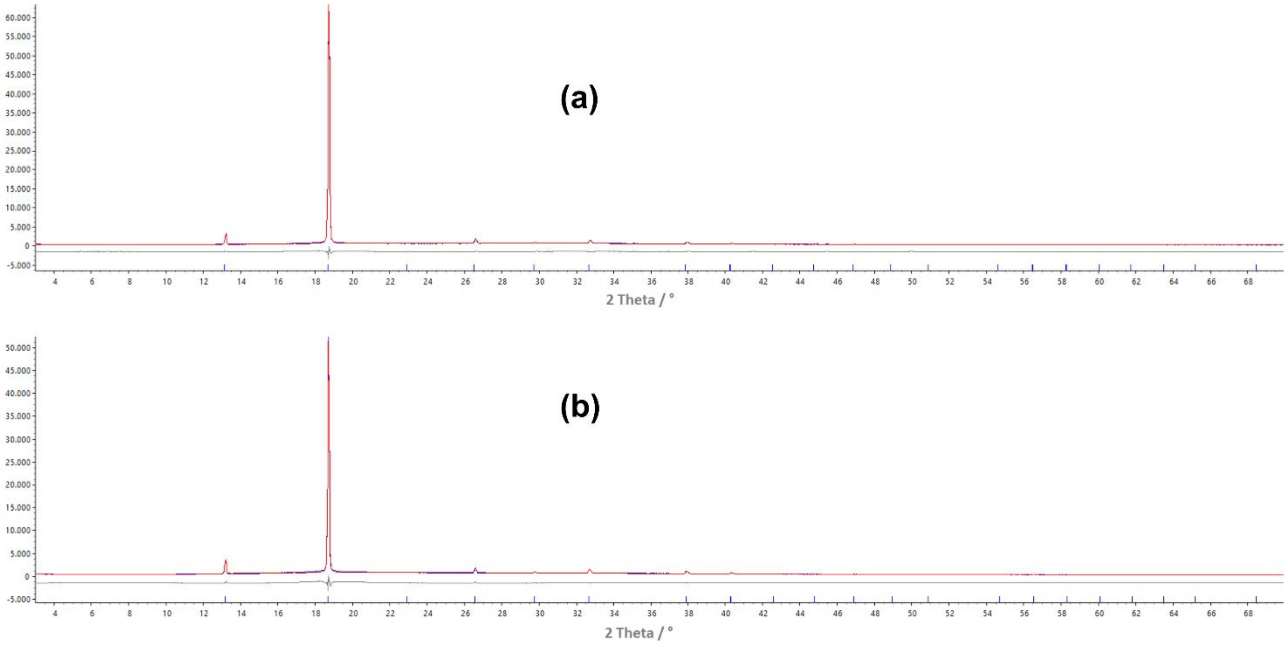

Figure SI5. Pawley refinemnts plots and FOMs for crystalline: (a) R-[QH]TFO, R_{wp} = 8.9%, GOF = 2.07; and (b) S/R-[QH]TFO, R_{wp} = 8.6%, GOF = 2.05. Experimental (blue), calculated (red) powder XRD patterns and difference profile (grey).

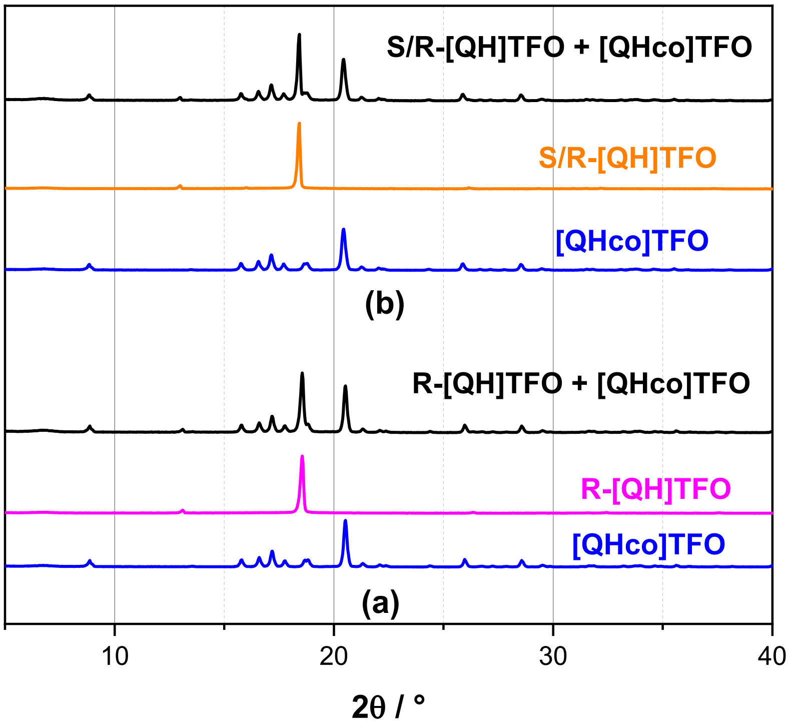

Figure SI6. Comparison between experimental powder XRD patterns recorded for polycrystalline: R-[QH]TFO, [QHco]TFO, and the attempted solid-solution R-$[QH]_{0.5}$·$[QHco]_{0.5}$TFO and resulting in a physical mixture; and (b) S/R-[QH]TFO, [QHco]TFO, and the attempted solid-solution S/R-$[QH]_{0.5}$·$[QHco]_{0.5}$TFO and resulting in a physical mixture.

φ-Scan images taken on a single crystal of R-[QH]TFO at RT and 250K

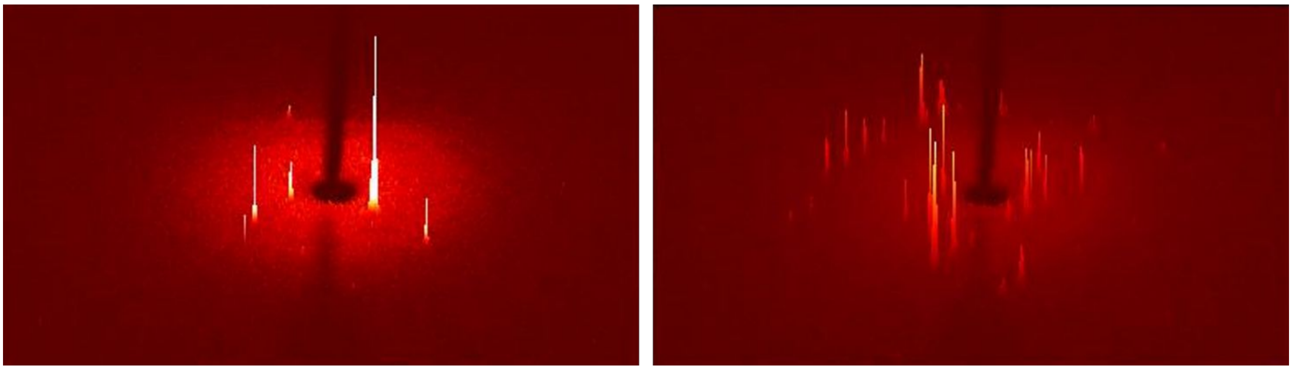

Figure SI7. 3D visualization of φ-Scan images captured from a single crystal of R-[QH]TFO at room temperature in its plastic phase (left) and after undergoing a cubic-to-orthorhombic transition at 250K (right).

Thermal analyses

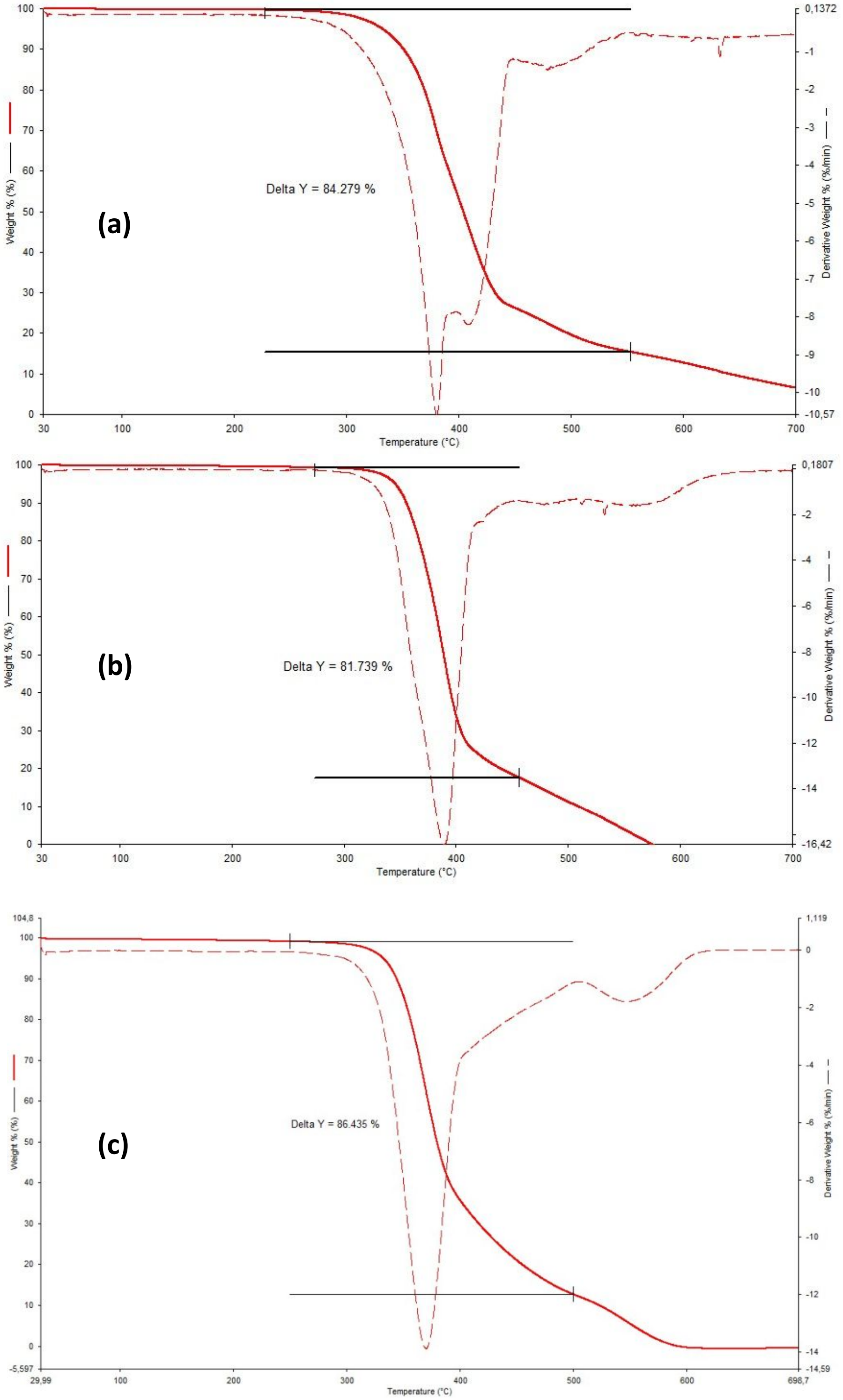

Figure SI8. Thermograms of polycrystalline powder samples: (a) [QHco]TFO, (b) R-[QH]TFO, and (c) S/R-[QH]TFO. Solid lines indicate weight loss, while dashed lines represent the first derivative of weight loss.

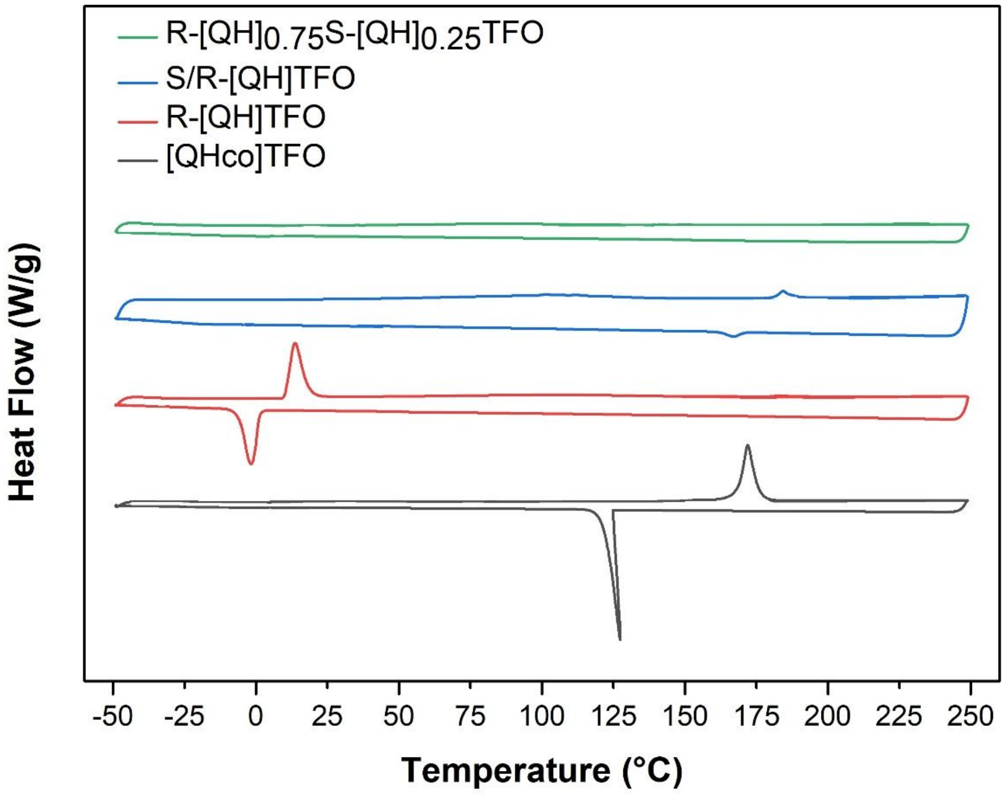

Figure SI9. DSC traces recorded on polycrystalline powder samples [QHco]TFO (black-line), R-[QH]TFO (red-line), S/R-[QH]TFO (blue-line), and R-$[QH]_{0.75}$·S-$[QH]_{0.25}$TFO.

Relative Change in Principal Axis Length for [QHco]TFO and R-[QH]TFO

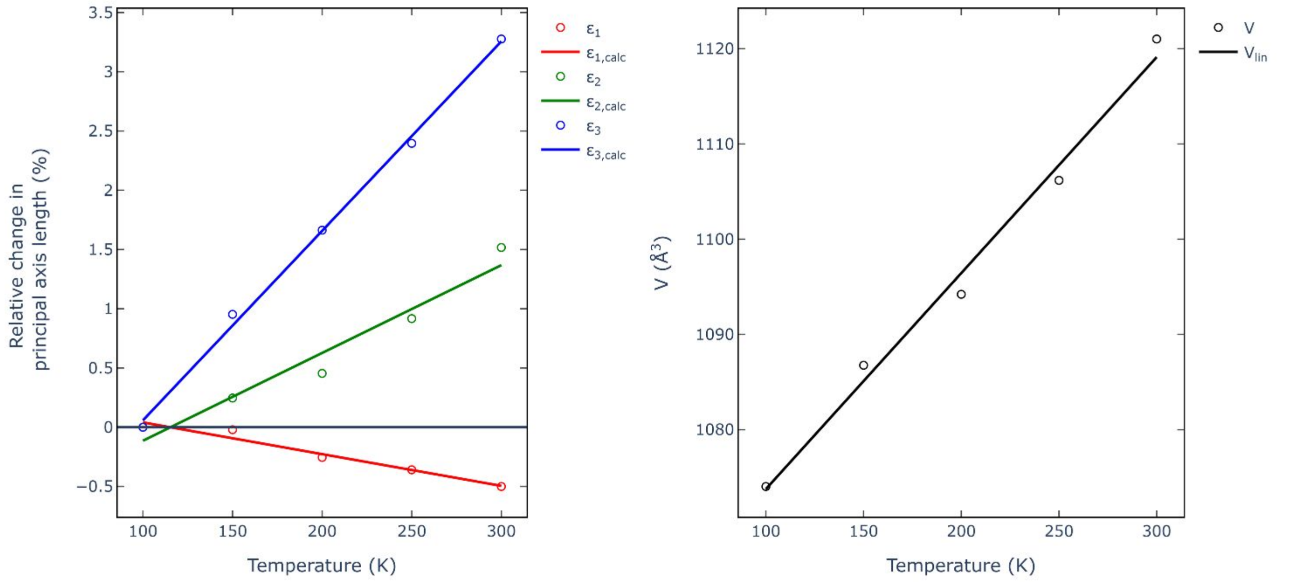

(a) **(b)**

Figure SI10. Relative change in principal axis length (a) and volume (b) for [QHco]TFO, calculated with PASCal.

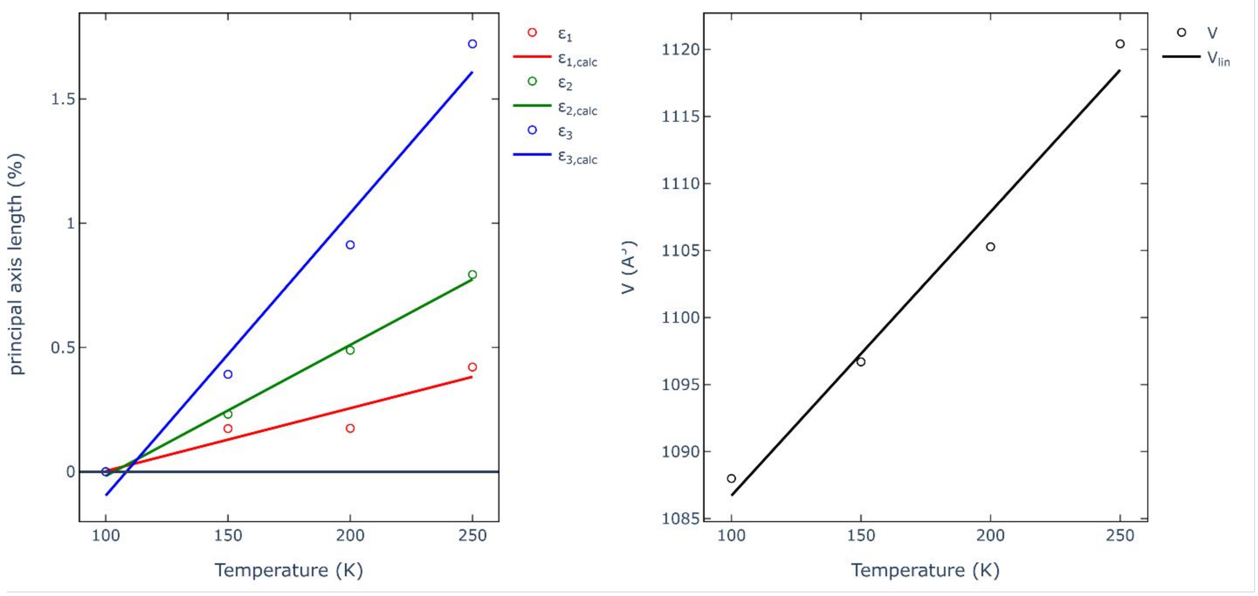

(a) **(b)**

Figure SI11. Relative change in principal axis length (a) and volume (b) for R-[QHco]TFO, calculated with PASCal.

Indicatrix Plots for [QHco]TFO and R-[QH]TFO

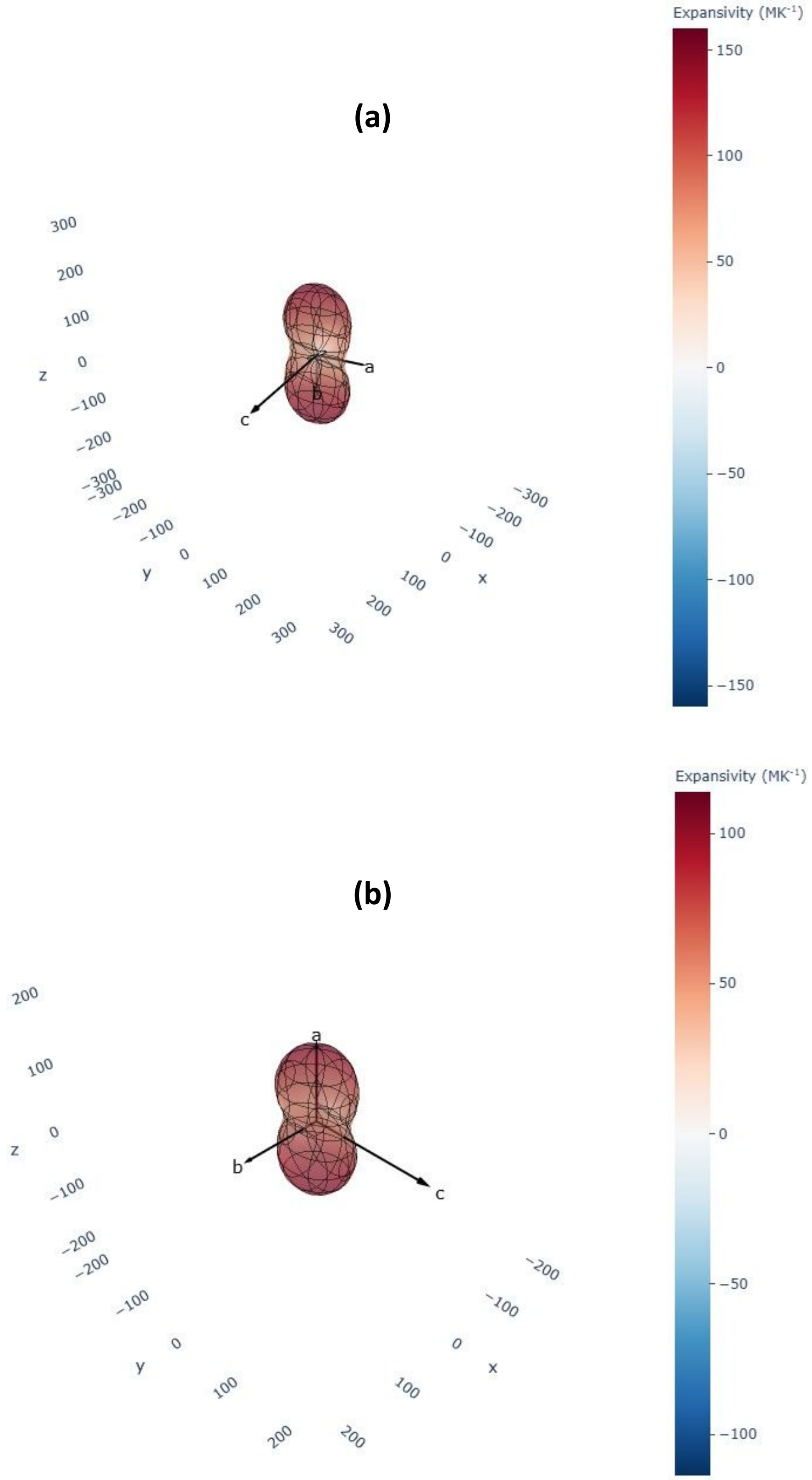

Figure SI12. Indicatrix plot (visual representation of principal axis thermal expansion coefficients) for: (a) [QHco]TFO and (b) R-[QH]TFO, derived from PASCal.

Raman Spectroscopy

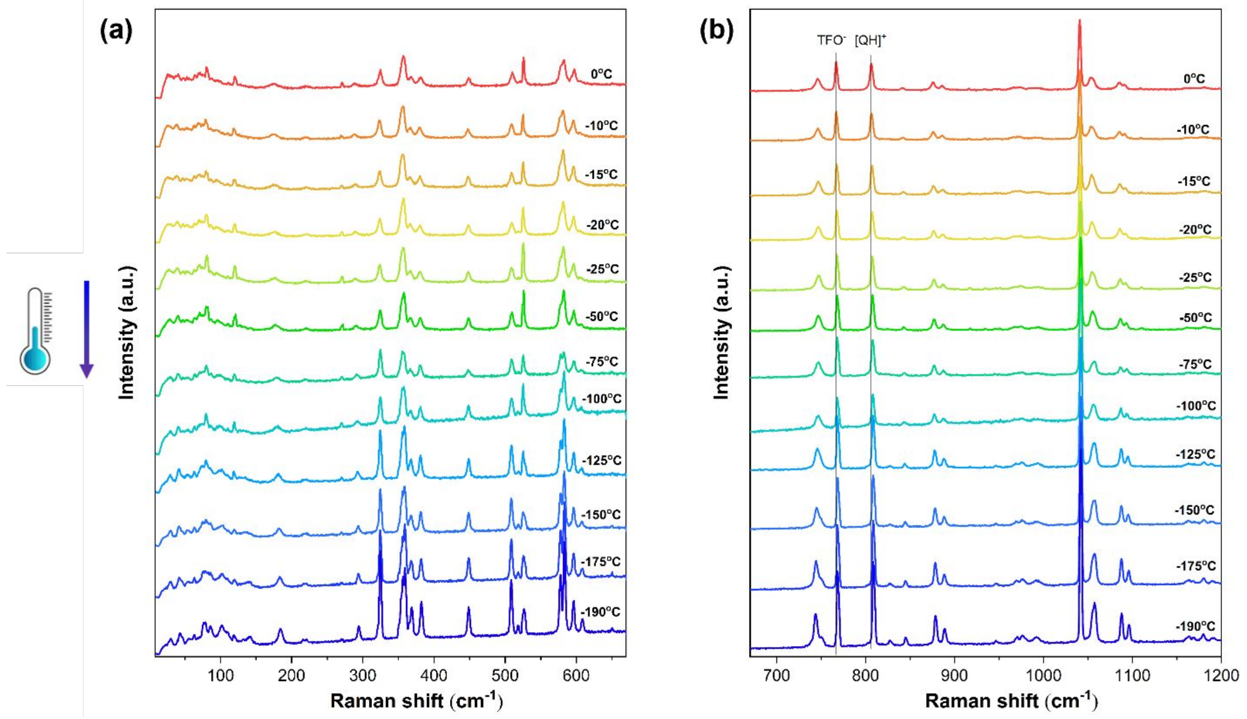

Figure SI13. Raman spectra for [QHco]TFO as a function of the temperature; (a) lattice phonon region (b) spectral range of the characteristic vibrational modes of the anion TFO^- and cation $[QH]^+$.

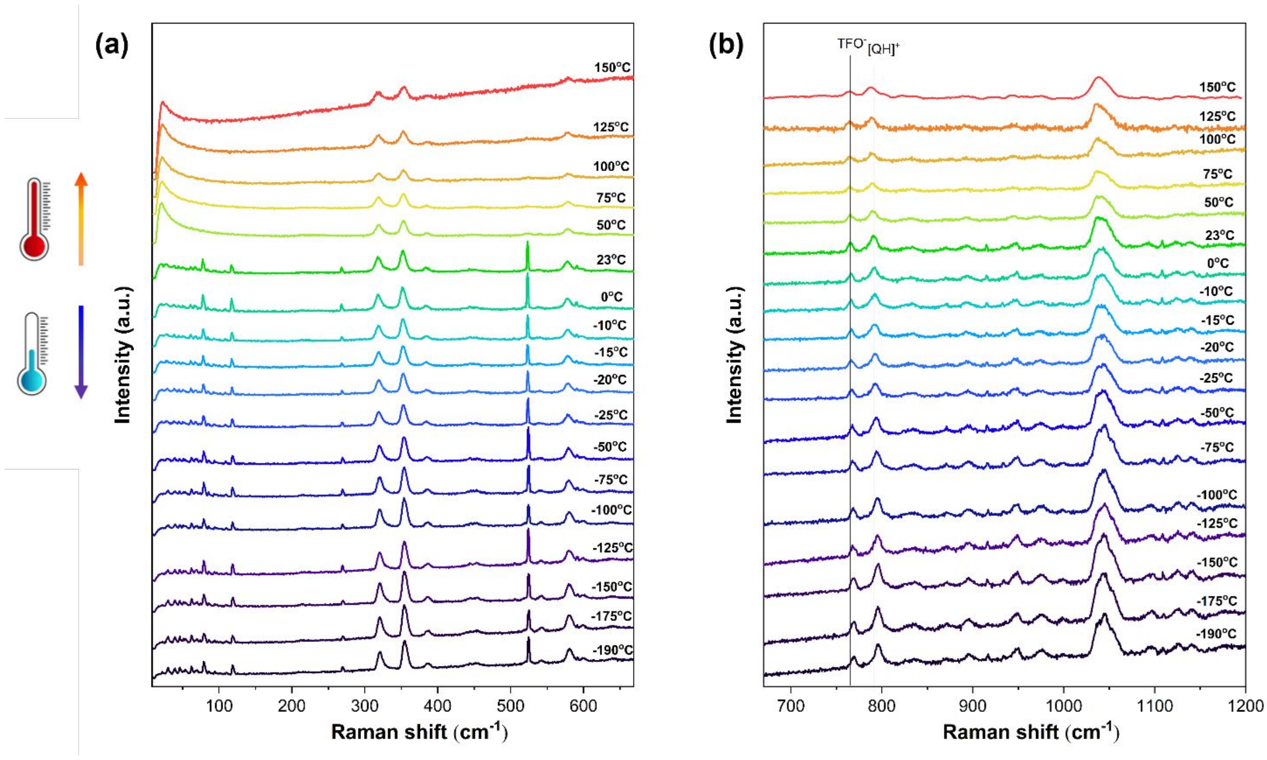

Figure SI14. VT Raman spectra for S/R-[QH]TFO as a function of the temperature; (a) lattice phonon region (b) spectral range of the characteristic vibrational modes of the anion TFO^- and cation $[QH]^+$.

Electrochemical Impedance Spectroscopy (Nyquist Plots)

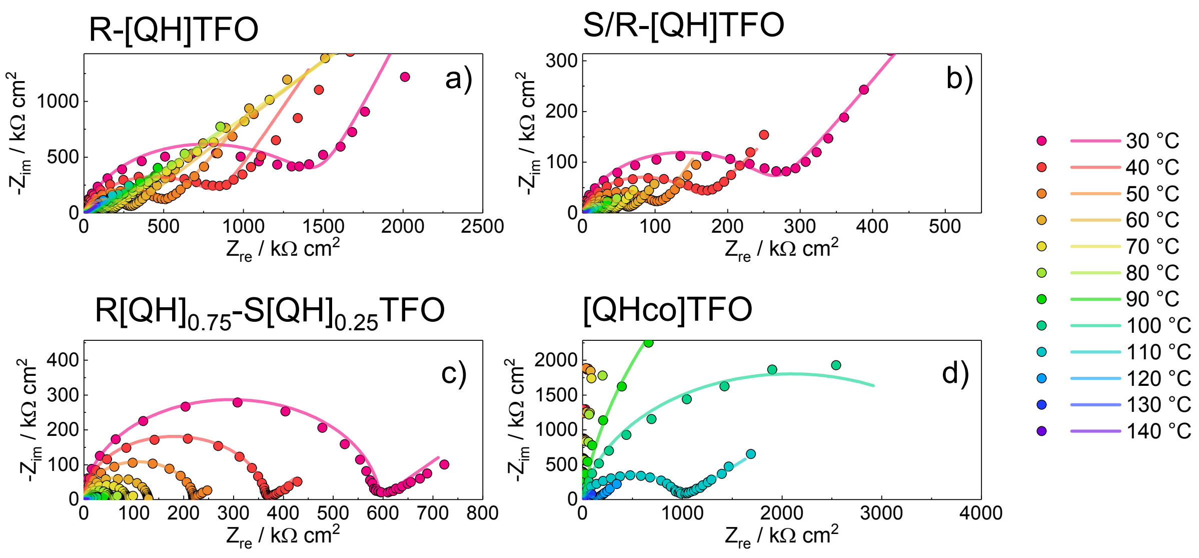

Figure SI15. Nyquist plots at different temperatures: (a) R[QH]TFO, (b) S/R[QH]TFO, (c) $R[QH]_{0.75}$-$S[QH]_{0.25}$TFO, and (d) [QHco]TFO. The experimental data are represented by the scatter points while the fit results are represented by the continuous line.
